# Hl48 modulates argonaute 2 to enhance RNA interference in ticks

**DOI:** 10.3389/fcimb.2026.1849245

**Published:** 2026-06-05

**Authors:** Qian Yao, Yongzhi Zhou, Jie Cao, Houshuang Zhang, Zedong Wang, Yanan Wang, Jinlin Zhou

**Affiliations:** 1 Shanghai Veterinary Research Institute, Chinese Academy of Agricultural Sciences, Shanghai, China; 2 Department of Infectious Diseases, Center of Infectious Diseases and Pathogen Biology, State Key Laboratory of Zoonotic Diseases, the First Hospital of Jilin University, Changchun, Jilin, China

**Keywords:** ago2, *Haemaphysalis longicornis*, RNA activation, RNA interference, tick control

## Abstract

**Background:**

Ticks are major vectors of human and animal pathogens, and the development of novel, species-specific control strategies is urgently needed. RNA interference (RNAi) holds promise as a targeted approach for tick control; however, its practical application has been limited by variable efficiency and an incomplete understanding of the underlying regulatory mechanisms in these arthropods.

**Methods:**

Using transcriptomic screening, molecular cloning, and functional genomics approaches, we identified and characterized Hl48, a previously uncharacterized ~48 kDa protein in *Haemaphysalis longicornis* (*H. longicornis*). Loss-of-function (RNAi) and gain-of-function (RNA activation, RNAa) strategies were employed to assess the role of Hl48 in RNAi regulation *in vivo*. Mechanistic investigations included dual-luciferase reporter assays, co-immunoprecipitation (Co-IP), molecular docking, site-directed mutagenesis, and surface plasmon resonance (SPR) analysis.

**Results:**

Hl48 expression was significantly upregulated in response to RNA virus infection or exogenous dsRNA stimulation, but not to protozoan infection, functionally linking it to the RNAi pathway. Silencing of Hl48 substantially impaired RNAi efficiency against multiple target genes, whereas its activation enhanced RNAi responsiveness both *in vivo* and *ex vivo*. Mechanistically, Hl48 regulates the RNAi pathway through a dual mode of action: it directly interacts with the PIWI domain of Argonaute 2 (AGO2), the catalytic core of the RNA-induced silencing complex (RISC), and concurrently promotes transcriptional upregulation of *ago2*. Key interaction residues (Thr28 and Glu93) were identified and validated by mutagenesis.

**Conclusion:**

These findings establish Hl48 as a key positive regulator of the RNAi pathway in ticks, revealing a previously unrecognized layer of RNAi control in arthropods. This work provides a mechanistic foundation for the development of enhanced RNA-based strategies for tick-borne disease control.

## Introduction

Ticks are obligate hematophagous arthropods that commonly parasitize vertebrates, including humans. Ticks serve as important vectors for a variety of zoonotic pathogens, posing a significant threat to global public health and animal husbandry (Ribeiro and Francischetti, 2003; Jongejan and Uilenberg, 2004; Dantas-Torres et al., 2012; Cabezas-Cruz et al., 2019). Current tick control primarily relies on chemical acaricides; this strategy faces increasing challenges, such as the development of resistance and environmental contamination (de la Fuente, 2018; Tabor et al., 2021). Consequently, there is an urgent need to develop novel, species-specific, and environmentally safe control strategies.

RNA-based gene regulation technology offers promising alternatives to chemical control (Setten et al., 2019). RNAi, which uses dsRNA to silence specific target genes, has emerged as a powerful tool with advantages in design flexibility and biosafety for non-target organisms (Baum et al., 2007). Compared with well-established insect models, the underlying mechanisms of RNAi in ticks remain unclear, significantly limiting its practical utility. Meanwhile, RNA activation (RNAa), an emerging technology that employs small activating RNAs (saRNAs) to upregulate gene expression at the transcriptional level, has shown potential in mammalian disease research (Li et al., 2006; Schwartz et al., 2008; Zheng et al., 2014; Musiała-Kierklo et al., 2025) but remains largely unexplored in ticks.

The RNAi pathway represents an evolutionarily conserved mechanism. Exogenous dsRNA is processed by Dicer into small interfering RNAs (siRNAs) that guide the RISC to degrade complementary mRNAs (Iwakawa and Tomari, 2022). AGO2 serves as the catalytic core of RISC and plays a pivotal role in this process (Meister, 2013). However, the efficiency of this pathway varies among species, especially the initial step of dsRNA uptake. In nematodes and mammals, systemic RNA interference-deficient (SID) family proteins are responsible for cellular dsRNA import (Cappelle et al., 2016; Wang R et al., 2024). In insects, clathrin-mediated endocytosis and scavenger receptor-like proteins have been implicated (Katoch et al., 2013; Xiao et al., 2015; Ye et al., 2021, 2023), and recent work in *Drosophila* identified the membrane-localized heat-shock protein Hsc70–4 as a specific dsRNA internalization factor (Fletcher et al., 2025). Despite the robust systemic RNAi response observed in ticks, the molecular mechanisms governing dsRNA remain largely unknown, creating a critical knowledge gap (de la Fuente and Kocan, 2022).

Selecting an appropriate model organism is essential for elucidating the mechanisms of RNAi functions in ticks. *H. longicornis* not only harbors a wide variety of tick-borne pathogens in China (Zhao et al., 2021) but also exhibits a strong systemic RNAi response, a trait whose molecular basis is less well understood than in insects or nematodes. In the present study, we identified and cloned a candidate gene, designated Hl48, whose expression is specifically induced by dsRNA and viral infection but not by other pathogenic stimuli, functionally linking it to the RNAi-related antiviral pathway. The conservation of this gene among hard ticks suggests an important functional role.

We employed complementary loss-of-function and gain-of-function approaches to determine whether Hl48 functionally regulates RNAi efficacy *in vivo*. Our results establish Hl48 as a modulator of the RNAi pathway in ticks, providing a foundation for further mechanistic analysis within the broader regulatory network of arthropod RNAi.

## Materials and methods

### Tick rearing and host animals

The parthenogenetic tick *H. longicornis* was maintained as described previously (Zhou et al., 2013). Adult ticks from the same cohort were used in all experiments. Kunming mice (KM, females, 6–8 weeks old, weighing 18–20 g) and BALB/c mice (females, 5–6 weeks old, weighing 18–20 g) were obtained from Shanghai Jiesijie Experimental Animal Co., Ltd (Shanghai, China). Kunming mice were used for molecular studies; BALB/c mice were used for parasite transmission and tick infection assays.

### Cell lines and culture

The *Ixodes ricinus* cell line IRE/CTVM19 (Bell-Sakyi et al., 2007) was supplied by the Tick Cell Biobank, University of Liverpool, and cultured at 30 °C in Leibovitz’s L-15 medium (Gibco, USA) supplemented with 20% FBS (Gibco, USA), 2 mM L-glutamine, 100 units/ml penicillin and 100 μg/ml streptomycin. HEK293T cells were maintained in DMEM (Gibco, USA) with 10% FBS at 37 °C under 5% CO_2_. Cells were passaged with 0.25% trypsin–EDTA at 80–90% confluency.

### Pathogen infection assays

#### *Babesia microti* infection

*B. microti* (provided by Shanghai Veterinary Research Institute) was maintained in BALB/c mice as described previously (Wu et al., 2017). Infected whole blood from donor mice was injected intraperitoneally into naïve BALB/c mice (female, 5–6 weeks old). Peripheral blood parasitemia was monitored daily by microscopic examination of Giemsa-stained blood smears. When parasitemia reached 1–3%, infected mice were used as a blood source for subsequent experiments or as feeding hosts for ticks. To infect ticks with *B. microti*, unfed adult *H. longicornis* was allowed to feed on the shaved dorsal skin of infected BALB/c mice. Engorged ticks were collected and infection was confirmed by Giemsa staining of tick hemolymph smears.

#### Alongshan virus infection

ALSV (Wang et al., 2019; Gui et al., 2026) was propagated in IRE/CTVM19 tick cells maintained at 28 °C in L-15 medium supplemented with 10% fetal bovine serum. For viral challenge, adult ticks were inoculated in the hemocoel with 5 × 10² TCID_50_ of ALSV per tick using a Nanoject II microinjection system (Drummond Scientific, Broomall, PA, USA). Injected ticks were then placed on KM mice for blood feeding. To investigate the effect of Hl48 on ALSV replication, IRE/CTVM19 cells were transfected with dsHl48 or dsLuc using RNAiMAX (Getico biotech, Shanghai, China) according to the manufacturer’s instructions. At 48 h post-transfection, cells were infected with ALSV. At 72 h post-infection, total RNA was extracted and ALSV genomic copy numbers were quantified by absolute quantification qRT-PCR using a standard curve as previously described (Gui et al., 2026). Results are expressed as viral copies per μL on a log_10_ scale. Three independent biological replicates were performed.

#### dsRNA microinjection

For dsRNA stimulation experiments, adult ticks were injected with 1 μL of dsRNA (1 μg/μL) or nuclease-free water (control) using the same microinjection system. After injection, ticks were allowed to feed on Kunming mice for blood feeding.

### Ethics statement

All animal procedures were approved by the Animal Ethics Committee of Shanghai Veterinary Research Institute (Approval No. SHVRI-20251212-03) and were performed in accordance with institutional guidelines.

### Sample collection, RNA extraction, and qRT-PCR

Samples included different developmental stages (unfed/engorged larvae, nymphs, and adults), tissues (salivary glands, ovaries, midgut, and hemolymph) from unfed adults, and tick cell lines. For each biological replicate, tissues or whole ticks from multiple individuals were pooled prior to RNA extraction to account for individual biological variability. Tissues were flash-frozen in liquid nitrogen and stored at −80 °C.

Total RNA was extracted with TRIzol (Invitrogen, USA). Spectrophotometry (NanoDrop 2000) and agarose gel electrophoresis were used to assess RNA quality. cDNA was synthesized from 1 μg of RNA using HiScript^®^ III RT SuperMix (Vazyme, China). qPCR was performed with ChamQ SYBR Master Mix (Vazyme, China) on a QuantStudio 5 system. The elongation factor-1α (Elf1α) served as the reference gene; expression was calculated using the 2^−^ΔΔCt method. Three biological and three technical replicates were run per assay. **Supplementary Tables 1** and **2** list the primers.

### Gene cloning and bioinformatic analysis

The Hl48 sequence was identified from an *H. longicornis* transcriptome database. The coding sequence (CDS) was amplified by conventional PCR using gene-specific primers and cloned for verification. The 5′ and 3′ untranslated regions were determined using 5′ and 3′ RACE, respectively (HiScript-TS 5′/3′ RACE Kit, Vazyme, China). The complete cDNA sequence was assembled and confirmed by sequencing. Protein homology was analyzed with NCBI BLASTP. SignalP 5.0 was used to predict signal peptides.

### dsRNA and saRNA synthesis and microinjection

The dsRNAs were designed from the CDS region of the Hl48 gene and synthesized *in vitro* using a T7 RNAi Transcription Kit (Vazyme, China). For saRNA design, a total of five candidate saRNA sequences (~20 nucleotides each) were designed and experimentally screened. Two candidates were designed targeting the 5′ UTR region of Hl48, which was obtained by RACE technology using the HiScript-TS RACE Kit (Vazyme, China). The other three candidates were designed based on predicted promoter regions within the 2,000 bp upstream of the Hl48 transcription start site, as annotated in the NCBI genomic sequence. All five candidate saRNAs were tested by qRT-PCR, and the two saRNAs targeting the 5′ UTR region demonstrated significantly higher activation efficiency and were therefore selected for subsequent experiments. The sequences of all candidate saRNAs are listed in **Supplementary Table 3**. Off-target potential was assessed by BLAST analysis against the NCBI nucleotide collection (nr/nt) database using blastn with a word size of 7. All non-target hits showed E values ≥ 1.5, indicating no significant off-target risk. The selected saRNAs were synthesized by Shanghai GeneBio Biotechnology.

Unfed adult ticks were injected with 0.5 μL of dsRNA or saRNA (1 μg/μL) into the hemocoel using a Nanoject II system. ds/saRNA targeting Luciferase (Luc) served as the negative control. After injection, ticks were maintained under standard rearing conditions for 24 hours (RNAi group) or 72 hours (RNAa group) before subsequent functional analyses.

### *Ex vivo* tissue isolation and dsRNA soaking treatment

Midgut and salivary gland tissues were dissected from adult *H. longicornis* under a sterile stereomicroscope. Tissues were placed in 24-well plates containing L-15 medium (Gibco, USA) at 500 µL per well and pre-incubated for 1 h at 28 °C to allow recovery. For dsRNA treatment, 1 µL of dsHl48 (1 µg/µL) was added to each well, with dsLuc at the same concentration used as a negative control. Tissues were incubated at 28 °C for 48 h. After incubation, tissues were gently washed three times with sterile PBS (pH 7.4), 5 min each, to remove residual dsRNA. Following washing, 500 µL of fresh L-15 medium was added to each well, followed by the addition of 1 µL dsECR (1 µg/µL). Tissues were incubated for an additional 48 h. Control tissues were treated with dsECR in the same manner. After the second incubation, tissues were collected, washed three times with PBS, snap-frozen in liquid nitrogen, and stored at −80 °C for subsequent RNA extraction and qRT-PCR analysis. All experiments were performed with three independent biological replicates (Grabowski and Kissinger, 2020).

### RNAi efficiency and phenotypic analysis

Two strategies were employed: “RNAi-of-RNAi” and “RNAi-of-RNAa” (Kwofie et al., 2023; Yoon et al., 2018). For RNAi-of-RNAi, ticks were first injected with dsHl48 or dsLuc; 24 h later, dsRNAs targeting Ecdysone receptor (ECR), Autophagy related 5 (ATG5), and Caspase8 were injected. After 24 h, the ticks were placed on rabbits for blood feeding. Engorged ticks were collected and recorded; non-engorged ticks were sampled at a standard time. For RNAi-of-RNAa, the ticks were pre-injected with saHl48 or saLuc; 72 h later, the same RNAi targeting was performed. Silencing efficiency was quantified using qRT-PCR. The engorgement rate and engorged weight were recorded. Each group consisted of thirty ticks, and three independent biological replicates were performed.

### Immunofluorescence localization

Tissues were fixed in 4% PFA, permeabilized with 0.5% Triton X-100, blocked with 5% skim milk, and incubated with mouse anti-Hl48 (1:1000) followed by Alexa Fluor 488-conjugated secondary antibody (1:500) (Invitrogen, USA). Nuclei were stained with DAPI. Cells were stained similarly, with additional phalloidin staining for the cytoskeleton. Images were acquired on a Zeiss LSM 880 (Carl Zeiss AG, Jena, Germany) confocal microscope and analyzed with ZEN software (Carl Zeiss AG) and ImageJ (National Institutes of Health, Bethesda, MD, USA).

### Dual-luciferase reporter assays

The *ago2* promoter region was cloned into the pGL3-Basic vector plasmid to generate the pGL3-*ago2*-promoter. The Hl48 CDS was cloned into the pCMV-Ha for overexpression. HEK293T cells seeded in 96-well plates were co-transfected with 500 ng of reporter, 500 ng of effector (or empty vector), and 10 ng of pRL-TK (internal control). After 48 h, luciferase activity was measured by Dual-Luciferase Reporter Assay System (Vazyme, China). Firefly luciferase activity was normalized to *Renilla* activity.

### Western blotting

Proteins were extracted with RIPA buffer, quantified using BCA, separated using SDS-PAGE (30 μg per lane), and transferred to PVDF membranes. The anti-Hl48 polyclonal antibody was generated in-house by immunizing female BALB/c mice with full-length recombinant Hl48 protein (421 amino acids) expressed and purified from *Escherichia coli*, and was used at a dilution of 1:1000. The anti-AGO2-PIWI polyclonal antibody was similarly generated in-house by immunizing female BALB/c mice with recombinant AGO2-PIWI protein (261 amino acids) expressed and purified from *Escherichia coli*, and was used at a dilution of 1:1000. The blots were also incubated with mouse anti-α-Tubulin (1:5000) (CST, USA), followed by HRP-conjugated secondary antibodies (1:5000) (Invitrogen, USA). Signals were detected with ECL and quantified with Image Lab software. Three independent replicates were performed.

### Co-immunoprecipitation

HEK293T cells were seeded in 6 cm dishes and transfected with 2 μg FLAG-AGO2-PIWI and HA-Hl48 plasmids using Lipofectamine 3000 (Invitrogen, USA) according to the manufacturer’s instructions. After 48 h, cells were lysed and incubated with anti-FLAG magnetic beads (LABLEAD, China). Bound proteins were eluted and analyzed using western blotting with anti-HA (CST, USA, 1:1000) and anti-FLAG antibodies (CST, USA, 1:1000). Transfection with an empty vector served as a control. Reverse Co-IP (using anti-HA beads) was also performed under the same conditions to confirm the interaction.

### Molecular docking and mutagenesis

The Hl48-AGO2-PIWI complex was predicted with AlphaFold3 (Abramson et al., 2024). Key interface residues were identified and evaluated using HADDOCK (Yan et al., 2020). Site-directed mutagenesis was performed to generate Hl48 point mutants. The mutant plasmids were transfected into HEK293T cells, and binding to AGO2-PIWI was assessed using Co-IP.

### SPR analysis

The interaction between Hl48 and the AGO2-PIWI domain was analyzed by Surface Plasmon Resonance (SPR) using a Biacore T200 instrument at 25 °C. Recombinant Hl48 protein was immobilized on a Series S Sensor Chip CMS via amine coupling according to the manufacturer’s instructions. A reference flow cell was activated and blocked without protein for background subtraction. A synthetic biotinylated peptide (23 aa) spanning the predicted binding region of the AGO2-PIWI domain was serially diluted in running buffer (10 mM HEPES pH 7.4, 150 mM NaCl, 0.005% Tween 20) to concentrations ranging from 0.5 to 16 μM and injected as an analyte at a flow rate of 30 μL·min^−1^, with association (150 s) and dissociation (300 s) phases. The surface was regenerated between cycles with 10 mM glycine–HCl (pH 2.0) for 60 s. Sensograms were reference-subtracted and globally fitted to a 1:1 binding model using Biacore T200 Evaluation Software (Cytiva, Uppsala, Sweden) to obtain the association rate (*k*_a_), the dissociation rate (*k*_d_), and the equilibrium dissociation constant (*K*_D_). Three independent experiments were performed (Yi et al., 2021).

### Statistical analysis

All data are presented as mean ± SD (n = 3 independent biological replicates). Statistical analyses were performed using GraphPad Prism 11 (GraphPad Software, Inc., San Diego, CA, USA). Comparisons between two groups were performed using unpaired Student’s t-test or Fisher’s exact test, as appropriate. Body weight data were compared using Mann-Whitney U test. Multiple-group comparisons were performed using one-way ANOVA followed by Tukey’s HSD *post hoc* test. Statistical significance was defined as *P < 0.05, **P < 0.01, ***P < 0.001, and ****P < 0.0001.

## Results

### Molecular cloning and sequence analysis of Hl48

**Figure 1:**
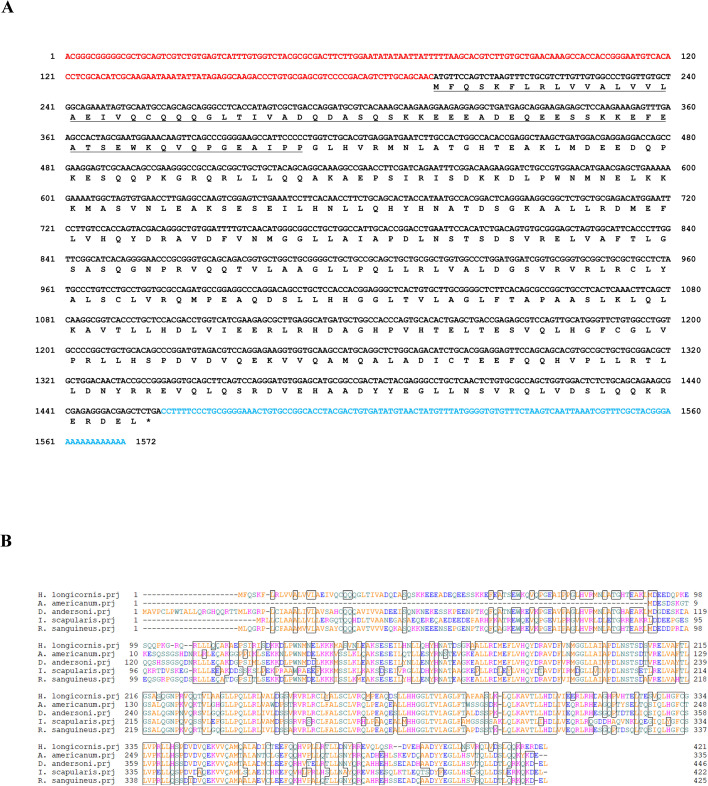
Identification of Hl48 as a conserved protein in ticks. **(A)** Schematic representation of the full-length Hl48 cDNA and its encoded protein. The 5′UTR is highlighted in red; the CDS is in black; and the 3′ UTR is in blue. The CDS encodes a protein of approximately 48 kDa. The predicted signal peptide is underlined. **(B)** Phylogenetic alignment of the Hl48 amino acid sequence confirms its identity as a conserved protein among various tick species.

To identify key molecules responsive to dsRNA stimulation in *H. longicornis*, we screened transcriptomic data and identified a previously uncharacterized gene that was significantly upregulated after virus infection (Wang Y et al., 2024) but showed no obvious change following parasitic infection (Chen et al., 2025). The \full-length cDNA sequence of this gene was obtained by molecular cloning (**Figure 1A**; **Supplementary Figure 1A**). The sequence contained a 1266-bp open reading frame encoding a protein of 421 amino acids with a predicted molecular weight of approximately 48 kDa, hence named Hl48 (GenBank accession number: PX753246.1). To further characterize Hl48, we obtained its complete 5’ UTR and 3’ UTR sequences using RACE technology (**Supplementary Figures 1B, C**), confirming the full-length structure of the transcript.

Phylogenetic analysis indicated that Hl48 is highly conserved among ticks. BLASTP alignment showed that the amino acid sequence of Hl48 shared 60–70% similarity with homologous proteins from *Dermacentor andersoni* (GenBank accession number: XP_050049127.1), *Amblyomma americanum* (GenBank accession number: KAK8781043.1), *Ixodes scapularis* (GenBank accession number: XP029849386.2), and *Rhipicephalus sanguineus* (GenBank accession number: XP037504179.1) (**Figure 1B**). This high degree of sequence conservation suggests that Hl48 likely performs an important biological function in ticks.

### Spatiotemporal expression and correlation with the RNAi pathway

**Figure 2:**
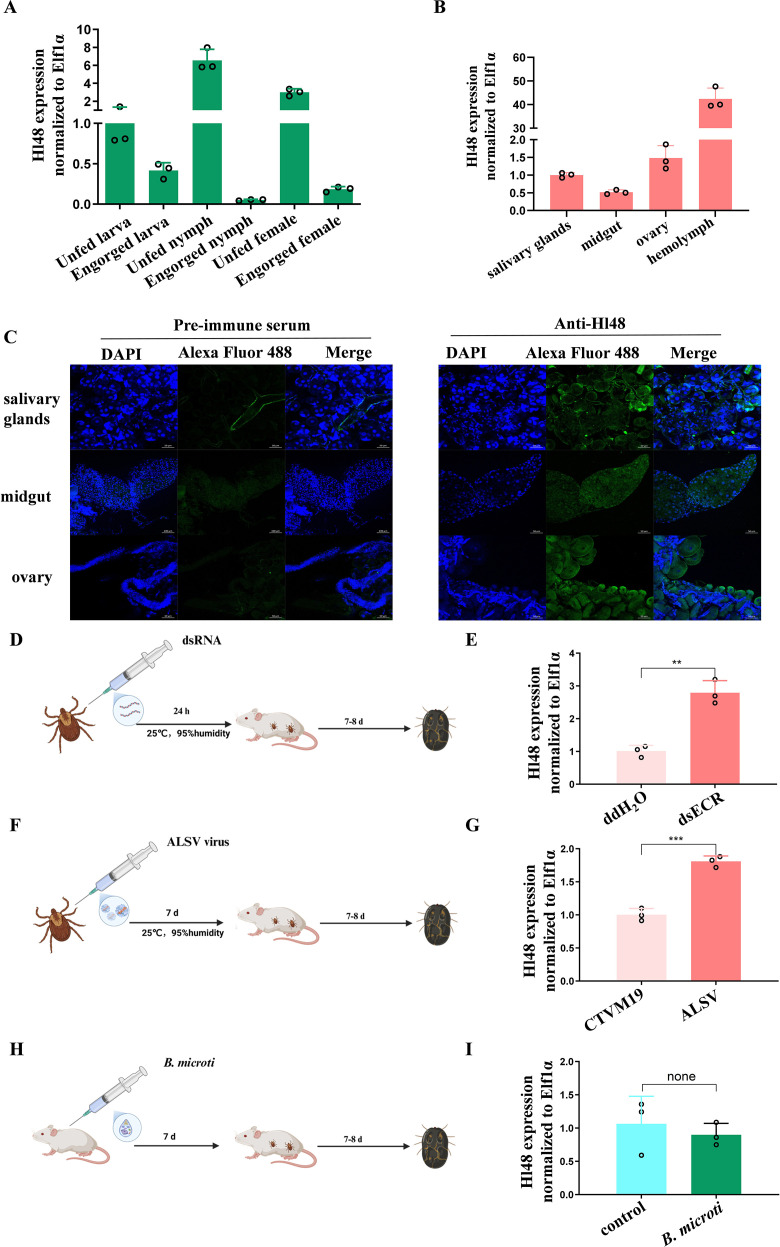
Spatiotemporal expression and pathogen-specific induction of Hl48. **(A)** Quantitative analysis of Hl48 mRNA expression across different developmental stages of *H. longicornis* whole ticks using qRT-PCR. **(B)** Quantitative analysis of Hl48 mRNA expression in different tissues of unfed adult *H. longicornis*. **(C)** Immunofluorescence staining showing the specific enrichment of Hl48 protein (green) in the salivary glands (SG), midgut (MG), and ovary (OV) of *H. longicornis*. Nuclei were counterstained with DAPI (blue). Scale bar, 50 μm. **(D, E)** Schematic of the dsRNA microinjection model and qRT-PCR analysis of Hl48 mRNA levels of whole ticks at indicated time points post-injection. **(F, G)** Schematic of the ALSV infection model and qRT-PCR analysis of Hl48 mRNA levels of whole ticks at indicated time points post-infection. **(H, I)** Schematic of the *B. microti* infection model and qRT-PCR analysis of Hl48 mRNA levels at indicated time points post-infection. Expression levels were normalized to ELF1α and are presented as mean ± SD (n = 3). *P < 0.05, **P < 0.01, and ***P < 0.001; NONE, not significant.

We systematically analyzed the spatiotemporal expression pattern of Hl48 in whole tick to characterize its biological function. The developmental stage expression profile revealed that Hl48 is expressed at all developmental stages of *H. longicornis*, but its expression is significantly suppressed after blood feeding (**Figure 2A**). Tissue distribution studies showed that Hl48 is specifically enriched in the salivary glands, midgut, ovary, and hemolymph, with its mRNA level in hemolymph being approximately 40−fold higher than that in the salivary glands (**Figure 2B****;** **Figure 2C**).

Notably, subcellular localization analysis revealed that Hl48 was distributed in both the cytoplasm and nucleus of HEK293T cells and IRE/CTVM19 cells (**Supplementary Figure 2**). This finding suggested its potential involvement in diverse functions across cellular compartments. To investigate the potential role of Hl48 in the tick RNAi pathway, we first analyzed its expression response under various stimulatory conditions. Three treatment models (dsRNA microinjection, ALSV infection, and *B. microti* infection (**Figures 2D, F, H**, **Supplementary Table 4**) were established. For dsRNA injection, ticks recovered for 24 h post−injection, allowing RNAi effects to be established before feeding. Ticks were then fed for 7–8 days and collected (**Figures 2D, E**). For ALSV infection, viral replication peaked at 7 days post−injection before feeding, after which ticks were fed for 7–8 days and collected (**Figures 2F, G**). For *B. microti* infection, ticks fed directly on infected mice for 7–8 days, because parasite acquisition requires ingestion of infected blood (**Figures 2H, I**). The results showed that Hl48 expression exhibited significant specificity: Hl48 mRNA levels were markedly upregulated following dsRNA injection and ALSV infection (**Figures 2E, G**), while no significant changes were observed under *B. microti* infection (**Figure 2I**). This result suggests that Hl48 expression responds to dsRNA and related viral challenges but not to protozoa infection, indicating its potential involvement in the RNAi pathway of ticks. To directly assess the functional consequence of Hl48 induction during viral infection, we performed knockdown experiments in the tick-derived cell line IRE/CTVM19. Transfection of dsHl48 significantly downregulated Hl48 expression compared with dsLuc controls, confirming effective gene silencing in tick cells (**Supplementary Figure 3A**). Upon ALSV challenge, dsHl48-treated cells exhibited significantly higher ALSV genomic copy numbers compared with dsLuc controls (**Supplementary Figure 3B**), indicating that knockdown of Hl48 impaired antiviral RNAi defense and resulted in enhanced viral replication. Taken together, these results demonstrate that Hl48 is not only induced by viral challenge but is also functionally required for restricting ALSV replication in tick cells, supporting its role as a key component of the antiviral RNAi pathway in *H. longicornis*.

### Hl48 is required for efficient RNAi *in vivo*

**Figure 3:**
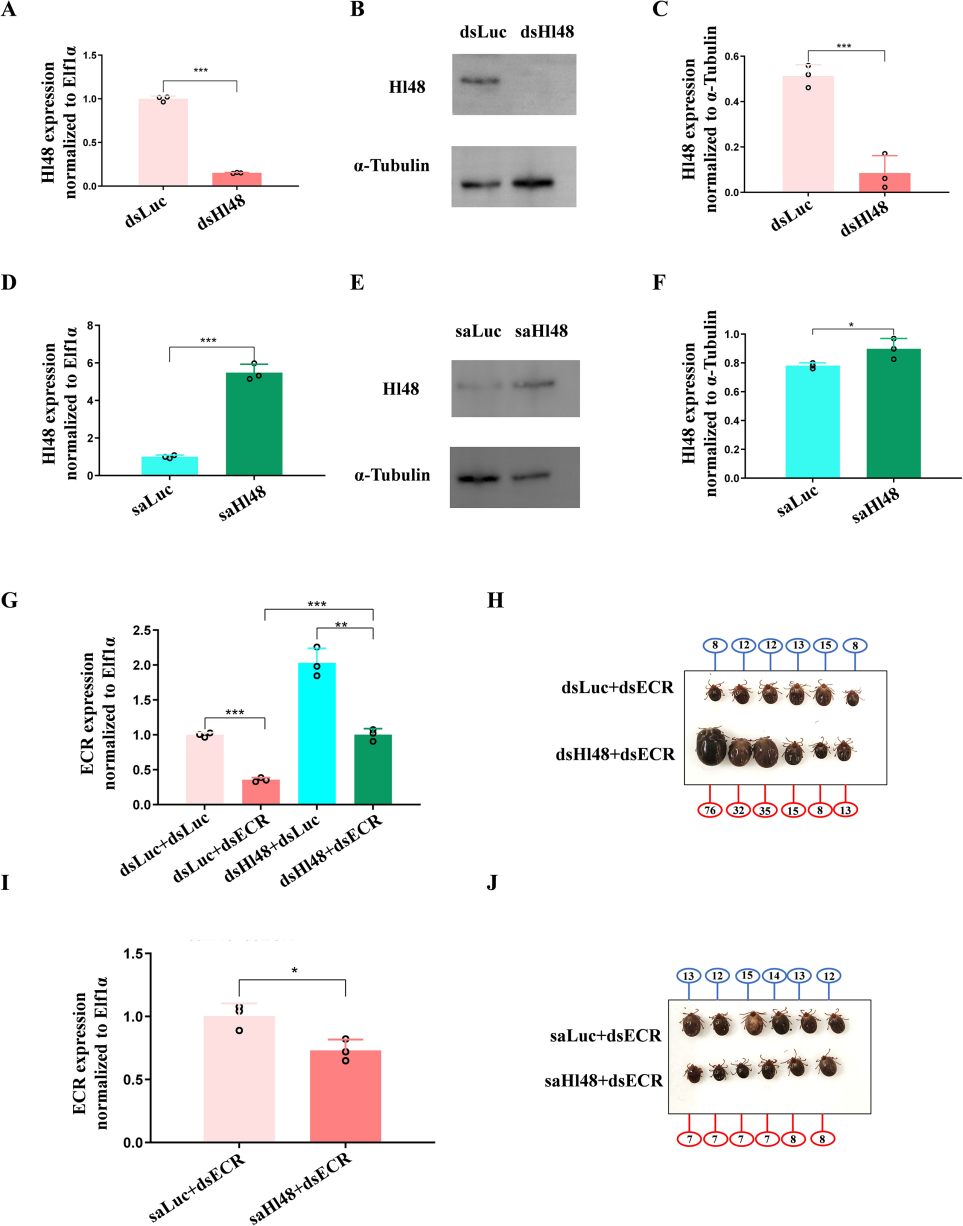
Hl48 functions as a master regulator of RNAi efficacy. **(A-C)** Validation of Hl48 interference efficiency. **(A)** qRT-PCR analysis of Hl48 mRNA levels in engorged adult whole ticks after dsHl48 injection. **(B)** Western blot analysis of Hl48 protein levels in engorged adult whole ticks after dsHl48 treatment. **(C)** corresponding quantification of Hl48 protein levels after dsHl48 treatment. **(D-F)** Validation of Hl48 activation efficiency. **(D)** qRT-PCR analysis of Hl48 mRNA levels in engorged whole adult ticks after saHl48 injection. **(E)** Western blot analysis of Hl48 protein levels in engorged adult whole ticks after saHl48 treatment. **(F)** corresponding quantification of Hl48 protein levels after saHl48 treatment. **(G)** qRT-PCR analysis of ECR mRNA levels in whole ticks pre-treated with dsLuc or dsHl48 followed by dsECR injection. **(H)** Representative images of engorged adult tick from the experiment in **(G)** Individual ticks within each group are marked with arrows or circles, and the numbers indicate engorged body weight in milligrams (mg). **(I)** qRT-PCR analysis of ECR mRNA levels in whole ticks following Hl48 activation and subsequent dsECR treatment. **(J)** Representative images of engorged adult ticks from the experiment in **(I)** Individual ticks within each group are marked with arrows or circles, and the numbers indicate engorged body weight in milligrams (mg). Data are presented as mean ± SD (n = 3). *P < 0.05, **P < 0.01, and ***P < 0.001; NONE, not significant.

We designed specific tools to manipulate Hl48 and investigated its regulatory function in the RNAi pathway. We employed dsRNA for RNAi knockdown and saRNA for RNAa−mediated activation. For saRNA design, five candidate sequences (~20 nucleotides each) were screened: two targeting the experimentally validated 5′ UTR region of Hl48 obtained by RACE, and three based on predicted promoter regions upstream of the transcription start site from the NCBI genomic sequence. The 5′ UTR-based saRNAs demonstrated significantly higher activation efficiency by qRT-PCR and were selected for subsequent experiments (**Supplementary Figure 4A****;** **Supplementary Table 3**). Both dsHl48 and saHl48 effectively modulated Hl48 expression at the transcriptional and protein levels (**Figures 3A–F**). Notably, neither dsHl48 nor saHl48 administration altered key biological parameters, including the engorgement rate or body weight, compared with their respective controls (dsLuc and saLuc) (**Supplementary Table 5**). This indicates that modulation of Hl48 did not broadly disrupt tick physiology, supporting the specificity of the subsequent functional analyses.

Using the established regulatory tools, we employed an “RNAi-of-RNAi” strategy to assess Hl48 function. The results showed that pre-knockdown of Hl48 significantly attenuated the efficacy of the RNAi against multiple target genes, including ECR (GenBank accession number: XM_077699443.1), ATG5 (GenBank accession number: MW116830.2), and Caspase8 (GenBank accession number: PP407944.1) (**Figure 3G****;** **Supplementary Figures 5A, E**). At the phenotypic level, Hl48 knockdown effectively reversed the negative effects, including the reduced engorgement rate and body weight loss, caused by silencing these target genes (**Figure 3H****;** **Supplementary Figures 5B, F**). The results indicate that Hl48 is essential for maintaining normal RNAi efficiency.

To obtain gain-of-function evidence, we designed and validated an saRNA of Hl48. The designed saRNA effectively induced Hl48 expression in a time- and dose-dependent manner, with maximal upregulation observed one day post-blood-feeding and at an optimal dose of 500 ng per tick (**Supplementary Figures 4B, C**). Using this tool, we implemented an innovative “RNAi-of-RNAa” strategy. In contrast to the RNAi-mediated knockdown results, pre-activation of Hl48 significantly enhanced the efficiency of RNAi against the same target genes (**Figure 3I**, **Supplementary Figures 5C, G**) and exacerbated the associated feeding defects and developmental abnormalities triggered by target gene silencing (**Figure 3J****;** **Supplementary Figures 5D, H**). Taken together, the evidence from both loss-of-function and gain-of-function approaches represented a coherent functional validation. Notably, the regulatory effect of Hl48 on RNAi efficiency was broad-spectrum, showing a consistent regulatory pattern across different target genes (ECR, ATG5, and Caspase8). A statistical analysis of multiple independent biological replicates of engorgement rate confirmed the stability and reproducibility of Hl48’s regulatory role across different RNAi target genes (**Supplementary Tables 6**, **7**).

**Figure 4:**
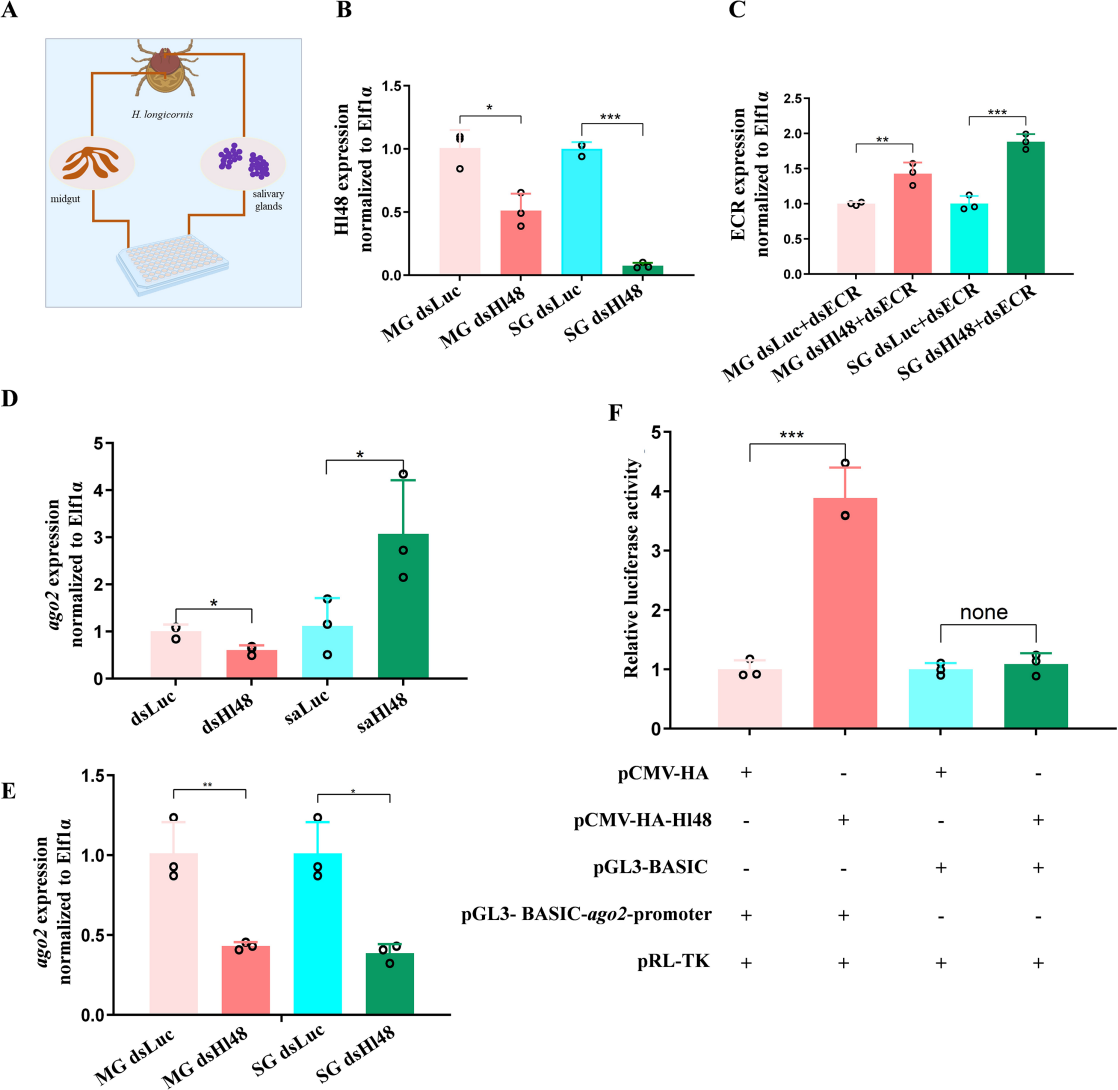
Hl48 transcriptionally regulates *ago2*. **(A)** Schematic diagram of the dissection of midgut and salivary glands from adult *H. longicornis*. **(B)** qRT-PCR validation of Hl48 knockdown efficiency in various tissues of *H. longicornis*. **(C)** qRT-PCR validation of dsECR interference efficiency in isolated salivary gland and midgut tissues pre-treated with dsHl48 or dsLuc. **(D)** Expression levels of *ago2* following Hl48 knockdown and upregulation in adult whole ticks. Data are presented as mean ± SD (n = 3). **(E)** qRT-PCR validation of *ago2* expression changes following Hl48 knockdown in cultured salivary glands and midguts. Data are presented as mean ± SD (n = 3). **(F)** Dual-luciferase assay of *ago2* promoter activity in HEK293T cells co-transfected with the pGL3-*ago2*-promoter reporter vector, pCMV-HA-Hl48 (or empty vector), and the pRL-TK *Renilla* reporter. Luciferase activities were measured 48 h post-transfection and are presented as Firefly/*Renilla* relative light units (RLU) normalized to the empty vector control. Data are presented as mean ± SD (n = 3). *P < 0.05, **P < 0.01, and ***P < 0.001; NONE, not significant.

We further isolated and cultured midgut and salivary gland tissues at the *ex vivo* tissue level (**Figure 4A**). Efficient Hl48 knockdown in these tissues was confirmed by qPCR (**Figure 4B****).** Subsequently, qPCR analysis revealed that the interference efficacy of dsECR against its target gene was significantly reduced following Hl48 knockdown (**Figure 4C**).

### Hl48 regulates AGO2 through transcriptional activation and direct protein interaction

To investigate whether Hl48 regulates the expression of *ago2*, a core component of RISC, we measured *ago2* transcript levels upon Hl48 modulation. qRT-PCR analysis at the whole-tick level showed that Hl48 knockdown significantly reduced *ago2* transcription, while Hl48 activation increased it (**Figure 4D**), indicating that *ago2* is a downstream target of Hl48. This finding was further validated *ex vivo*: Hl48 knockdown in cultured salivary gland and midgut tissues also suppressed *ago2* expression (**Figure 4E**).

To further dissect the regulatory mechanism, we cloned the promoter region of the *ago2* gene (**Supplementary Figures 6A, B**) and analyzed it using a dual-luciferase reporter assay system. The results showed that overexpression of Hl48 in HEK293T cells significantly activated the *ago2* promoter, increasing luciferase activity approximately fourfold (**Figure 4F**). This result directly demonstrates that Hl48 can regulate *ago2* expression at the transcriptional level.

**Figure 5:**
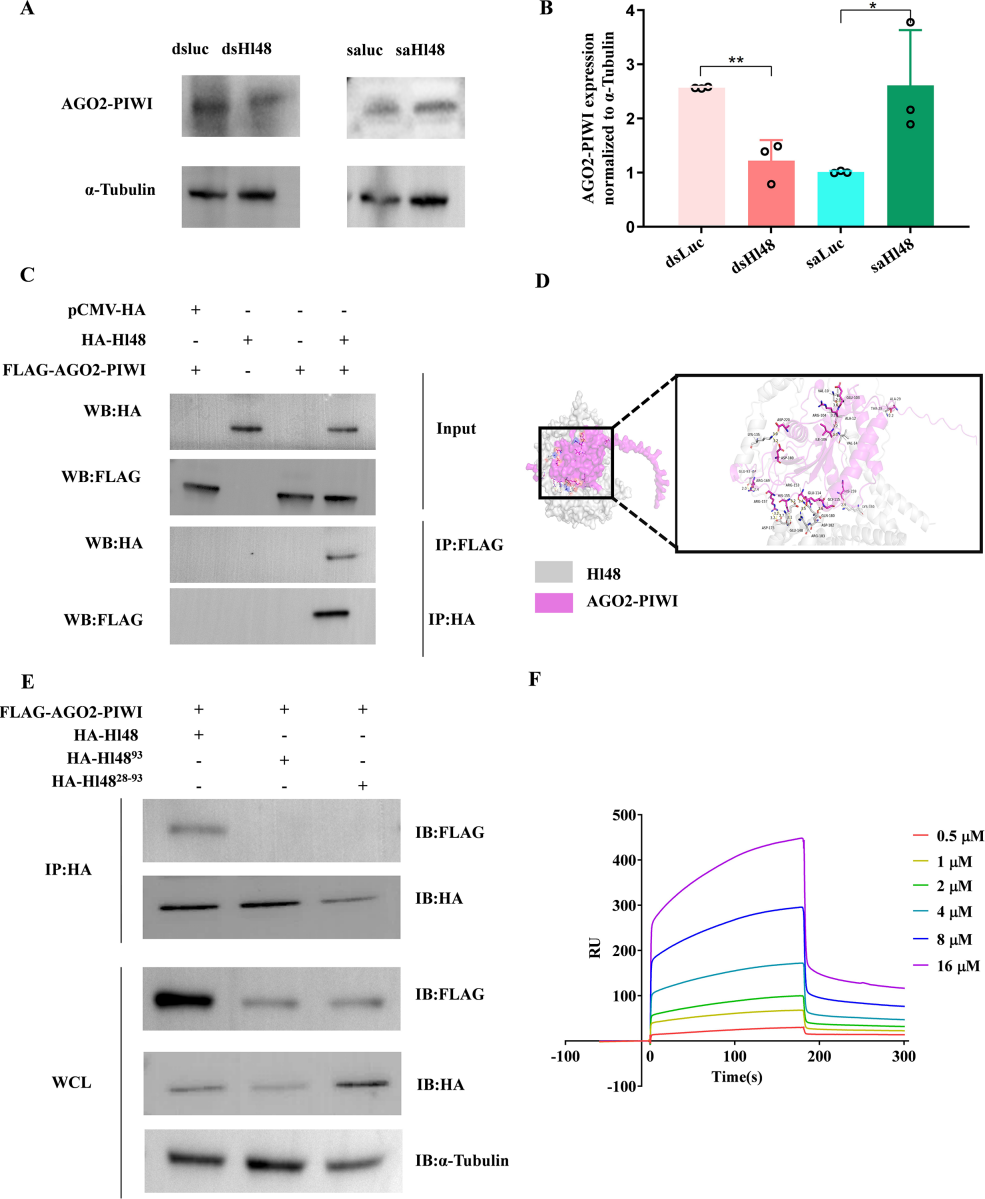
Hl48 directly interacts with the AGO2-PIWI domain. **(A)** Western blot analysis showing AGO2-PIWI protein levels in engorged adult whole ticks treated with dsLuc/dsHl48 (left) or saLuc/saHl48 (right). α-Tubulin was used as a loading control. **(B)** corresponding quantification analysis of AGO2-PIWI expression following Hl48 modulation in **(A)**. **(C)** Co-IP assay confirming the direct protein–protein interaction between HA-tagged Hl48 and FLAG -tagged AGO2-PIWI domain in HEK293T cells. Cell lysates were immunoprecipitated with anti-FLAG beads and immunoblotted with anti-Ha and anti- FLAG antibodies. **(D)** Molecular docking model predicting the key binding residues at the Hl48-AGO2-PIWI interface. The AGO2-PIWI domain is shown in red, and Hl48 is shown in gray. Key interacting residues are highlighted and labeled. **(E)** Co-IP assay validating that single or double mutations of the predicted key residues in Hl48 impair its interaction with AGO2-PIWI. Wild-type (WT) or mutant HA-Hl48 constructs (Single mutation HA-Hl48^93^ and double mutation HA-Hl48^28-93^) were co-expressed with FLAG-AGO2-PIWI in HEK293T cells, and Co-IP was performed as in **(C)**. **(F)** Kinetic fitting of the interaction between Hl48 protein and AGO2-PIWI peptide by SPR analysis. Data are presented as mean ± SD (n = 3). *P < 0.05 and **P < 0.01.

After demonstrating that Hl48 regulates *ago2* at the transcriptional level, we sought to determine whether Hl48 also affects AGO2 protein stability. Western blot analysis using an antibody recognizing the PIWI domain of AGO2 showed that Hl48 expression levels correlated positively with AGO2-PIWI abundance: Hl48 knockdown decreased, and Hl48 activation increased, AGO2-PIWI protein levels (**Figures 5A, B**).

We performed Co-IP assays to verify the direct interaction between Hl48 and AGO2-PIWI. The results demonstrated specific binding between HA-tagged Hl48 and the FLAG -tagged AGO2-PIWI in HEK293T cells (**Figure 5C**), indicating that Hl48 directly interacts with the catalytic core of AGO2.

To delineate the molecular details of the interaction interface, we employed computational molecular docking to model the Hl48-AGO2-PIWI complex. The predicted model identified several key amino acid residues potentially involved in hydrogen bonding and hydrophobic interactions (**Figure 5D****;** **Supplementary Table 8**). Based on the predicted binding interface (**Supplementary Table 8**), we selected Hl48 residues THR-28 and GLU-93 for mutagenesis because they are in close proximity to corresponding AGO2-PIWI residues. Single mutation (HA-Hl48^93^) and double mutation (HA-Hl48^28−93^) both impaired the interaction (**Figure 5E**), confirming their critical role in binding (**Figure 5E**), confirming their role in binding and supporting the reliability of the predicted interface. Multiple sequence alignment further revealed that both residues are partially conserved across hard tick species, with greater conservation observed in species more closely related to *H. longicornis*, suggesting that this interaction mechanism may be broadly applicable within Ixodidae but may differ in more distantly related tick lineages (**Figure 1B**). We conducted SPR analysis to corroborate this interaction in a cell-free system and obtain quantitative binding data. The assay used a purified Hl48 protein immobilized on a sensor chip and a synthetic peptide encompassing the key predicted binding region of the AGO2-PIWI domain as the analyte. The SPR assays confirmed a direct and measurable interaction between the two molecules (**Figure 5F**). The binding profile yielded a dissociation constant (K_D_) of 2.64 µM. Notably, the interaction was characterized by rapid association (k_a_ = 6.47 × 10^2^ M^−1^s^−1^) and much slower dissociation (k_d_ = 1.71 × 10^−3^ s^−1^), resulting in a complex with a long half-life of approximately 6.8 minutes (**Supplementary Table 9**). This provides precise biophysical evidence for the formation of a stable complex.

**Figure 6:**
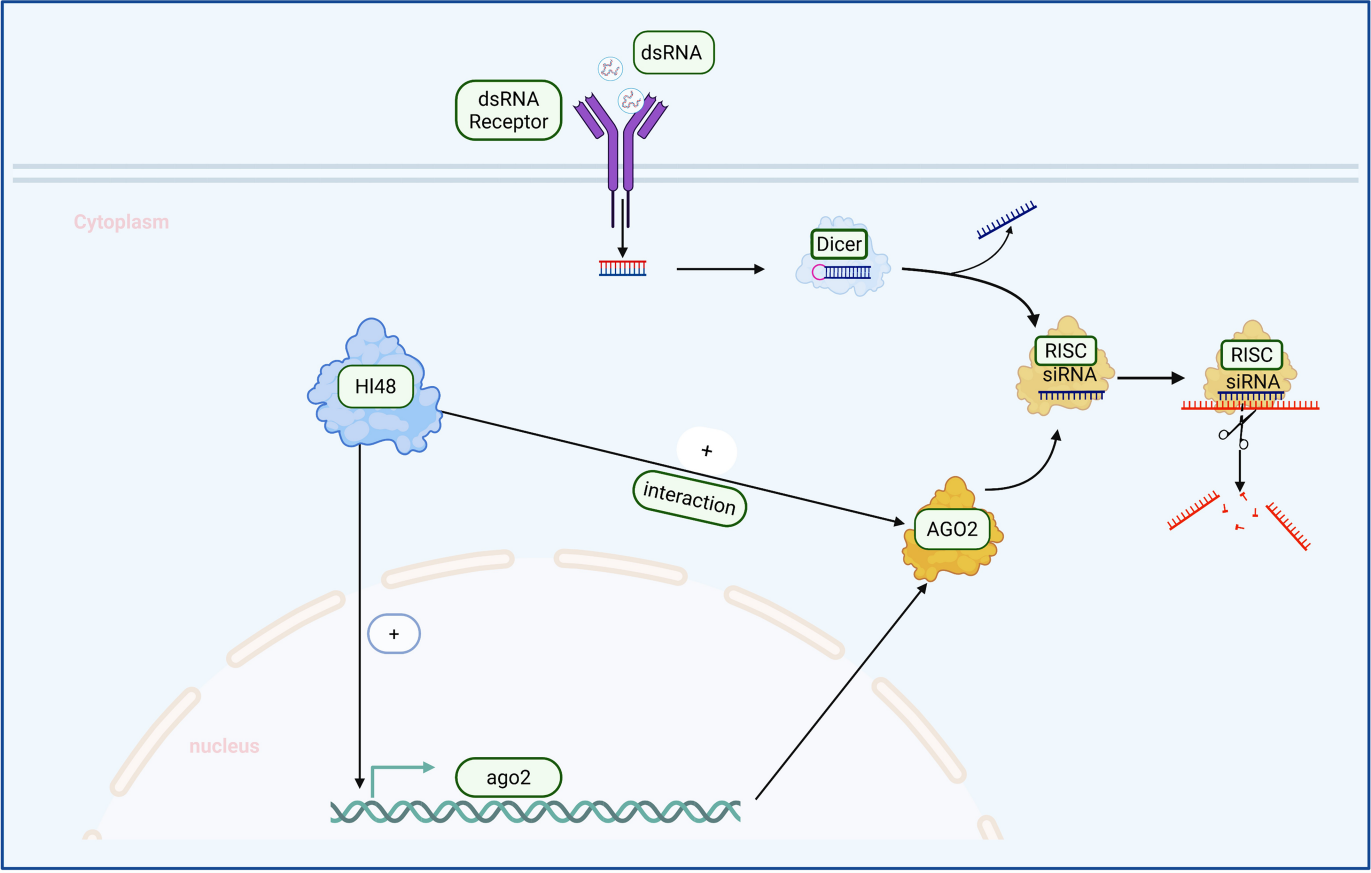
Graphical abstract summarizing the role of Hl48 in RNAi regulation of ticks. The Hl48 protein functions through a dual mechanism: it transcriptionally upregulates the expression of the core RNAi gene ago2 and directly interacts with the AGO2 protein, likely stabilizing it or facilitating its function. This synergistic action amplifies the overall efficiency of the RNAi pathway against target genes.

Collectively, the convergence of evidence from Co-IP and mutagenesis establishes Hl48 as a direct binding partner of the AGO2-PIWI domain. Based on these findings, we propose a model in which Hl48 serves as a key regulator that enhances RNAi efficacy in tick (**Figure 6**).

## Discussion

The operation of the RNAi mechanism exhibits a high degree of species diversity among arthropods. While its core components are conserved, the upstream recognition, uptake, and regulatory networks are characteristic of taxa. The Hl48 molecule identified in *H. longicornis* in this study offers a new perspective on RNAi regulatory networks in ticks. Compared with insects and mammals, the tick RNAi system displays several distinct evolutionary adaptive features.

This study marks the first successful application of RNAa technology in a tick research system. RNAa technology has been applied in mammalian cells and disease models for gain-of-function gene studies (Musiała-Kierklo et al., 2025), but its application in arthropods, especially non-insect groups, has been extremely limited. We established an “RNAi-of-RNAa” research strategy, enabling gain-of-function studies of target genes in live ticks. This methodological breakthrough not only provides a new technical tool for functional genomics research in ticks but also enables verification of Hl48’s biological functions from both loss-of-function and gain-of-function dimensions, significantly enhancing the reliability of our conclusions. At the same time, the method offers a novel and powerful tool for functional genomics research in ticks and other arthropods that are difficult to transfect or genetically manipulate. Compared with traditional methods such as overexpression (which may cause artifacts due to ectopic or non-physiological expression levels) or gene editing (a technology less well developed in ticks; while CRISPR-Cas9 has been successfully used in some tick species (Sharma et al., 2022), a robust toolkit for *H. longicornis* is not yet available), RNAa enables specific activation of endogenous genes, more closely mimicking the physiological state. Together with RNAi, which retains practical advantages including technical simplicity, reversibility, and the ability to silence multiple genes simultaneously, these approaches provide complementary loss-of-function and gain-of-function strategies for rapid and reliable functional genomics research in ticks. Notably, during the design of saRNAs targeting Hl48, candidates based on genomic predictions showed no significant activation, whereas those designed from the experimentally validated 5′ UTR sequence obtained by RACE demonstrated significantly higher efficiency, likely reflecting inaccuracies in the current *H. longicornis* genome annotation. This highlights the importance of using experimentally validated transcript sequences rather than solely relying on genomic predictions for saRNA design, particularly in non-model organisms with incomplete genome assemblies. At the level of systemic RNAi regulation, our study is the first to demonstrate that Hl48 regulates the RNAi pathway through a dual mechanism. On the one hand, Hl48 directly interacts with the PIWI domain of AGO2. This mechanism bears interesting functional similarities to auxiliary proteins such as TRBP in mammals (Chendrimada et al., 2005). However, sequence alignment showed no homology between Hl48 and these proteins, representing a classic case of functionally convergent evolution. This highlights how AGO2, as the effector center of RNAi, has independently evolved diverse protein partners in different species to optimize its function. On the other hand, and uniquely, Hl48 can also positively regulate the transcription of the *ago2* gene. This dual mode of regulating both the upstream (transcriptional) and downstream (protein interaction) aspects of a single pathway is extremely rare among currently reported RNAi auxiliary factors. It may represent an efficient positive feedback or synergistic amplification mechanism: sensing dsRNA induces Hl48 expression. The expression of Hl48 both enhances *ago2* transcription to expand the production capacity of the core machinery and, through direct binding, stabilizes the AGO2 protein and potentially optimizes its function, thereby synergistically enhancing the overall responsiveness and silencing efficiency of the entire RNAi pathway. Molecular docking identified Thr28 and Glu93 as key residues mediating the Hl48–AGO2-PIWI interaction, which was further validated by single (Hl48-93) and double (Hl48-28-93) point mutant experiments. Multiple sequence alignment revealed that both residues are partially conserved across hard tick species, with greater conservation observed in species more closely related to *H. longicornis*, suggesting that this interaction mechanism may be broadly applicable within Ixodidae but may differ in more distantly related tick lineages.

In addition to its functional roles in the RNAi pathway, the subcellular localization of Hl48 warrants discussion. Although SignalP analysis predicted the presence of a signal peptide in Hl48, immunofluorescence results demonstrated that Hl48 localizes to both the cytoplasm and nucleus. This apparent discrepancy may be attributed to signal peptide misprediction, as SignalP is known to generate false positives for certain protein classes (Garcion et al., 2021). Alternatively, the signal peptide may be cleaved co-translationally, with the mature protein subsequently redirected to the cytoplasm and nucleus through interaction with retention factors (Arnoys and Wang, 2007). Notably, the observed dual localization is functionally consistent with the demonstrated roles of Hl48 in interacting with cytoplasmic AGO2 and regulating nuclear ago2 transcription, suggesting that cytoplasmic and nuclear residence reflects its biological function rather than a mis localization artifact.From a functional perspective, the “RNAi-of-RNAi” and “RNAi-of-RNAa” strategies employed in this study systematically verify the effect of Hl48 on the RNAi efficacy of multiple key genes. These results are consistent with studies in other tick species: for example, interfering with homologous genes such as Dicer or AGO2 in *Rhipicephalus microplus* leads to significant developmental abnormalities and reduced fecundity (Kurscheid et al., 2009). Particularly noteworthy is the high expression pattern of Hl48 in salivary glands and ovaries. This feature aligns closely with the biological characteristics of ticks as disease vectors, suggesting its potentially important roles in pathogen transmission and vertical transmission.

Additionally, we found that Hl48 is also highly expressed in hemolymph, with mRNA levels approximately 40−fold higher than those in the salivary glands (**Figure 2B**). This raises the possibility that Hl48 may function in tick immune cells, although further studies are needed to define its precise role in this compartment. Notably, Hl48 orthologs are highly conserved among hard ticks (**Figure 1B**); however, their functions have not been experimentally investigated in other species. Future studies should address whether they play similar roles in RNAi regulation.

From an applied perspective, the discovery of Hl48 offers a dual opportunity for developing a new generation of tick control technologies. First, Hl48 represents a highly promising and specific target. Designing activators such as saRNA against Hl48 could potentiate the tick’s own RNAi efficiency, thereby enhancing the efficacy of co-administered dsRNA-based insecticides or strengthening RNAi-mediated physiological responses. Second, the RNAa technology validated in this study serves as a prototype for a potential “gene drive”-like control tool. In the future, one could design saRNAs targeting essential genes for tick survival or reproduction. Abnormally activating the expression of these genes could have lethal or sterilizing effects, representing a novel conceptual approach distinct from traditional silencing strategies (RNAi).

Although this study has made significant progress, many questions remain. For example, what is the specific molecular pathway by which Hl48 mediates intracellular dsRNA transport? Does it intersect with endosomal systems, autophagy pathways, or other known nucleic acid-sensing pathways? Furthermore, the precise molecular mechanism of RNAa in tick cells (e.g., whether it involves chromatin remodeling or promoter demethylation) and its universality across other tick species and genes are key to the broad applicability of this method. Finally, and perhaps most intriguingly, there remains the question of whether the expression and function of Hl48 are regulated or exploited by tick-borne pathogens under natural infection conditions; this should be investigated in future studies. This knowledge would connect fundamental RNAi research with the practical control of tick-borne diseases.

In conclusion, this study systematically identified a novel RNAi regulatory hub, Hl48, in *H. longicornis*. The Hl48 gene profoundly influences the efficacy of the RNAi pathway in ticks through dual mechanisms: promoting intracellular dsRNA utilization and synergistically activating AGO2. This discovery not only enriches our understanding of the diversity of RNAi mechanisms in arthropods, revealing unique adaptive evolutionary strategies in ticks, but also provides valuable resources and ideas at both the methodological (RNAa application) and translational (innovative targets and strategies) levels. Future in-depth research focusing on Hl48 and its associated network will continue to advance the fields of tick biology and tick-borne disease control.

## Data Availability

The raw data supporting the conclusions of this article will be made available by the authors, without undue reservation.
