# Supplementary material for: Hl48 modulates argonaute 2 to enhance RNA interference in ticks
Source: Front Cell Infect Microbiol. 2026 Jun 5;16:1849245. doi: 10.3389/fcimb.2026.1849245 (PMC13280550; doi:10.3389/fcimb.2026.1849245)
Supplement: Supplementary file 1 [file DataSheet1.zip › supplementary figure.pdf]

SUPPLEMENTARY FIGURE

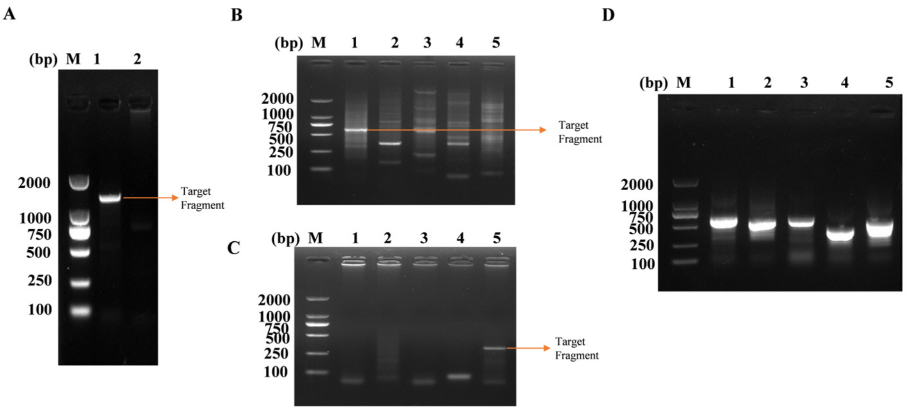

Figure S1. Determination of the Full-Length Hl48 Transcript. (A)Electrophoretic analysis confirming the successful amplification of the Hl48 ORF. M. DNA marker; 1. Hl48; 2. negative control. (B)Validation of the 5' UTR sequence obtained by 5' RACE PCR with gene-specific primers. M. DNA marker; 1. 5'GSP-1; 2. 5'GSP-2; 3. 5'GSP-3; 4. 5'GSP-4; 5. 5'GSP-5. (C)Validation of the 3' UTR sequence obtained by 3' RACE PCR with gene-specific primers. M. DNA marker; 1. 3'GSP-1; 2. 3'GSP-2; 3. 3'GSP-3; 4. 3'GSP-4; 5. 3'GSP-5. (D) Agarose gel electrophoresis of synthesized dsRNAs. M. DNA marker; 1. dsLuc; 2. dsHl48; 3. dsECR; 4. dsATG5; 5. dsCaspase8.

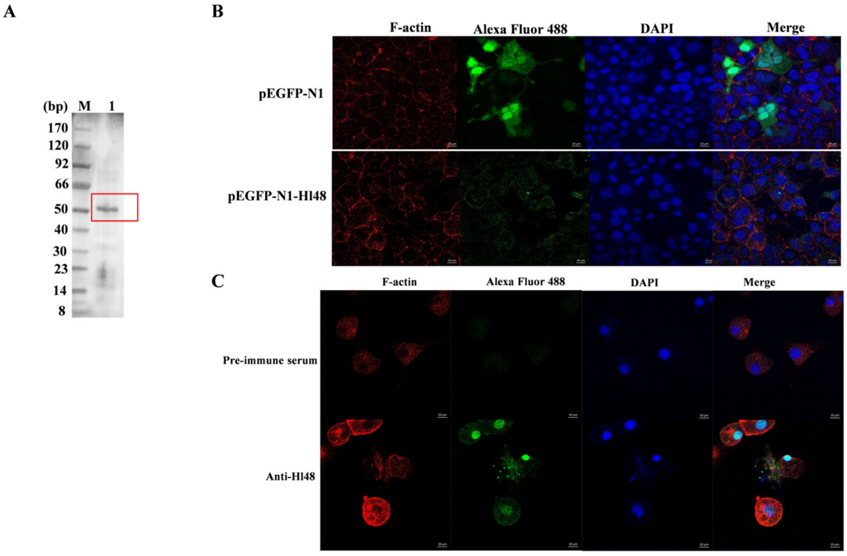

Figure S2. Validation of Custom Hl48 Antibody and Subcellular localization of Hl48 in HEK293T cells. (A)Verification of the anti-Hl48 antibody specificity using Western blot. (B-C) Subcellular localization of Hl48 in HEK293T cells (B) and IRE/CTVM19 cells (C). Cells were transfected with a Hl48-expressing plasmid for 48 hours and subjected to immunofluorescence staining. Hl48 (green) shows a predominantly localized in the cytoplasm and nucleus. F-actin (red) and DAPI (blue) mark the cytoskeleton and nuclei, respectively. Scale bar, 10 μm.

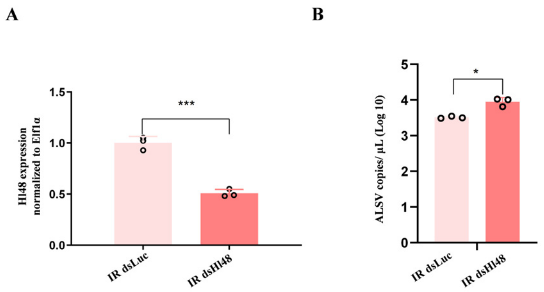

Figure S3. Interference of Hl48 in tick cells impairs antiviral RNAi and promotes ALSV replication. (A) RNAi efficiency of dsHl48 in cell line IRE/CTVM19. Cells were transfected with dsHl48 or dsLuc using RNAiMAX, and Hl48 mRNA levels were quantified by qRT-PCR at 48 h post-transfection. Data are presented as mean ± SD (n = 3). ***p < 0.001. (B) ALSV genomic copy numbers in IRE/CTVM19 cells following Hl48 knockdown. Cells transfected with dsHl48 or dsLuc were infected with ALSV at 48 h post-transfection. Viral RNA was extracted and copy numbers were determined by absolute quantification qRT-PCR using a standard curve. Results are expressed as ALSV copies per µL and presented on a $\log_{10}$ scale. Data are presented as mean ± SD (n = 3). *p < 0.05.

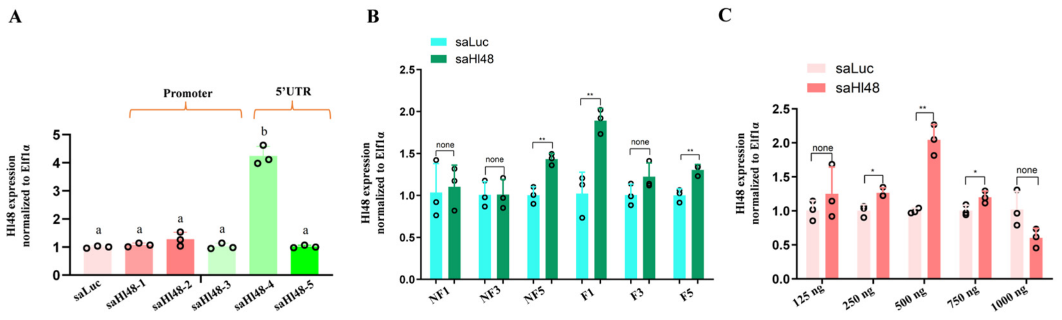

Figure S4. validation and optimization of saHl48. (A) Screening of candidate saRNA sequences targeting Hl48. Four candidate saRNAs were designed using two strategies: two based on predicted promoter regions upstream of the transcription start site (saRNA-1 and saRNA-2) and three based on the experimentally validated 5′ UTR sequence obtained by RACE (saRNA-3, saRNA-4 and saRNA-5). Hl48 mRNA levels were quantified by qRT-PCR in engorged adult whole ticks after saHl48 injection. (B) Time-course analysis of Hl48 mRNA levels in unfed and fed adult ticks after injection with saHl48. NF, unattached; F, attached. (C) Determination of the optimal injection dose for saHl48 by evaluating Hl48 expression in whole tick after administration of increasing amounts of saRNA. Data are presented as mean ± SD (n = 3). one-way ANOVA followed by Tukey's HSD post hoc test (A); Student's t-test (B, C). *p < 0.05, **p < 0.01, NONE, not significant.

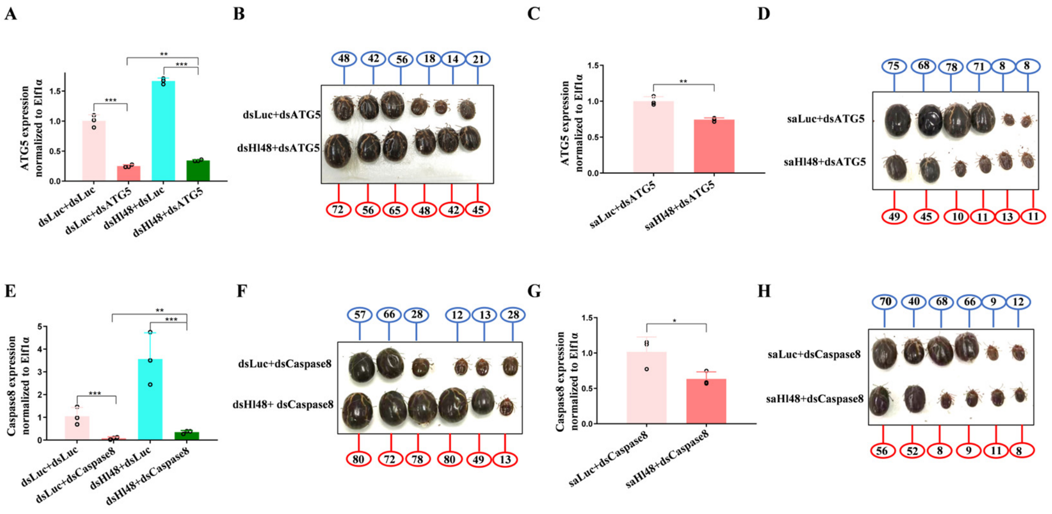

Figure S5. Hl48 modulates RNAi efficiency of dsATG5 and dsCaspase8 in *H. longicornis*. (A) qRT-PCR analysis of ATG5 mRNA levels in whole ticks pre-treated with dsLuc or dsHl48 followed by dsATG5 injection. (B) Representative images of engorged ticks from dsATG5 RNAi-of-RNAi experiments; numbers indicate individual body weight (mg). (C) qRT-PCR analysis of ATG5 mRNA levels in whole ticks following Hl48 activation and subsequent dsATG5 treatment. (D) Representative images of engorged ticks from dsATG5 RNAi-of-RNAa experiments. (E) qRT-PCR analysis of Caspase8 mRNA levels in whole ticks pre-treated with dsLuc or dsHl48 followed by dsCaspase8 injection. (F) Representative images of engorged ticks from dsCaspase8 RNAi-of-RNAi experiments; numbers indicate individual body weight (mg). (G) qRT-PCR analysis of Caspase8 mRNA levels in whole ticks following Hl48 activation and subsequent dsCaspase8 treatment. (H) Representative images of engorged ticks from dsCaspase8 RNAi-of-RNAa experiments. Data are presented as mean ± SD (n = 3). *p < 0.05, **p < 0.01, ***p < 0.001.

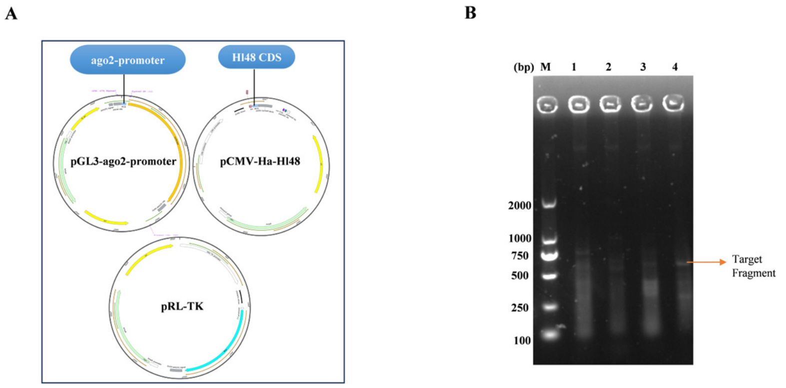

Figure S6. Molecular Identification and Transcriptional Regulation Analysis of ago2. (A) Schematic representation of dual-luciferase reporter constructs for assessing ago2 promoter activity. (B) Agarose gel electrophoresis of ago2 promoter using gene-specific primers. M. DNA marker; 1. 5’GSP-1; 2. 5’GSP-2; 3. 5’GSP-3; 4. 5’GSP-4.
