# Supplementary material for: Hl48 modulates argonaute 2 to enhance RNA interference in ticks
Source: Front Cell Infect Microbiol. 2026 Jun 5;16:1849245. doi: 10.3389/fcimb.2026.1849245 (PMC13280550; doi:10.3389/fcimb.2026.1849245)
Supplement: Supplementary file 1 [file DataSheet1.zip › supplementary figure.docx]

**SUPPLEMENTARY FIGURE**


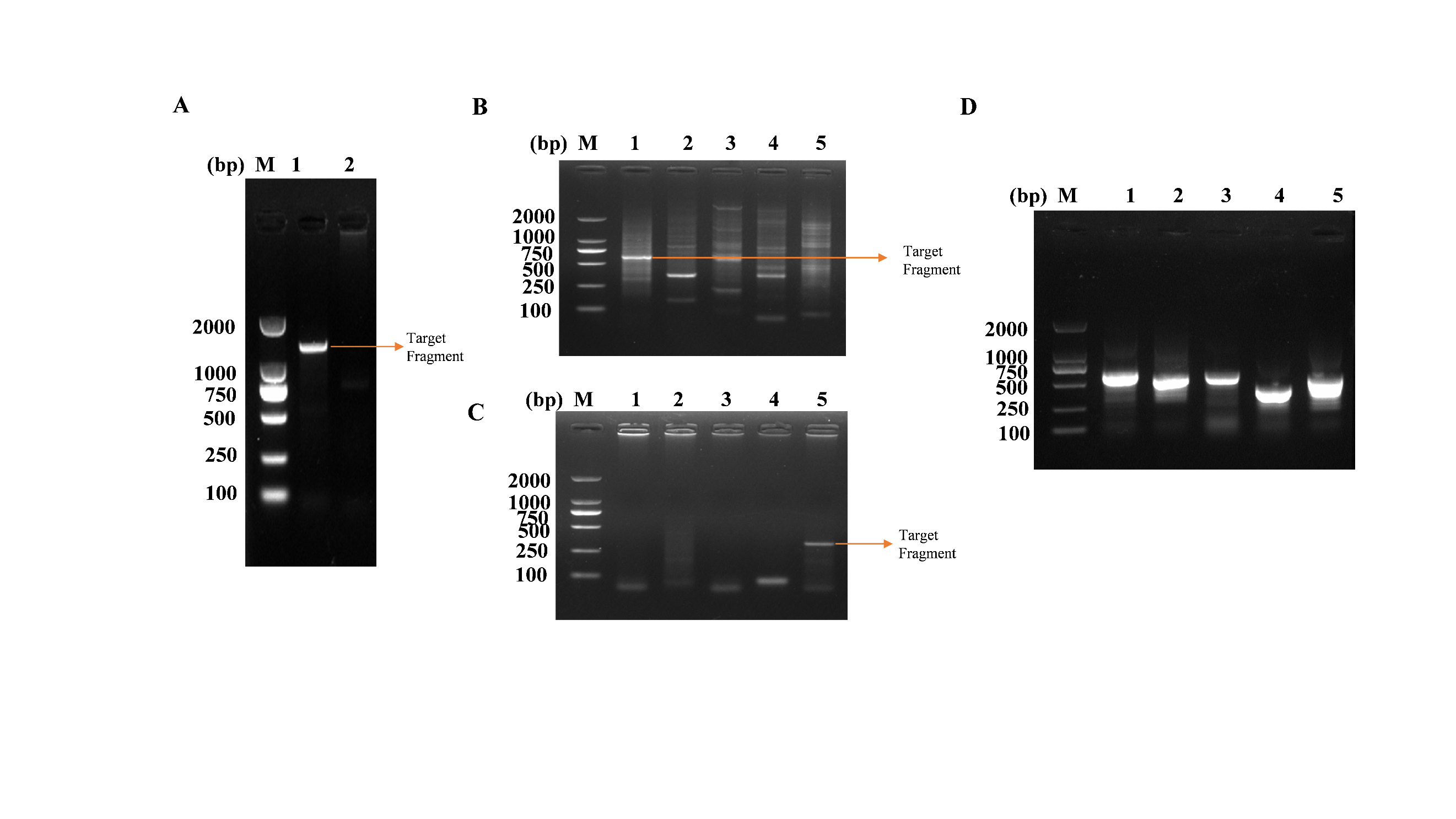


**Figure S1. Determination of the Full-Length Hl48 Transcript.** (A)Electrophoretic analysis confirming the successful amplification of the Hl48 ORF. M. DNA marker; 1. Hl48; 2. negative control. (B)Validation of the 5' UTR sequence obtained by 5' RACE PCR with gene-specific primers. M. DNA marker; 1. 5’GSP-1; 2. 5’GSP-2; 3. 5’GSP-3; 4. 5’GSP-4; 5. 5’GSP-5. (C)Validation of the 3' UTR sequence obtained by 3' RACE PCR with gene-specific primers. M. DNA marker; 1. 3’GSP-1; 2. 3’GSP-2; 3. 3’GSP-3; 4. 3’GSP-4; 5. 3’GSP-5. (D) Agarose gel electrophoresis of synthesized dsRNAs. M. DNA marker; 1. dsLuc; 2. dsHl48; 3. dsECR; 4. dsATG5; 5. dsCaspase8.


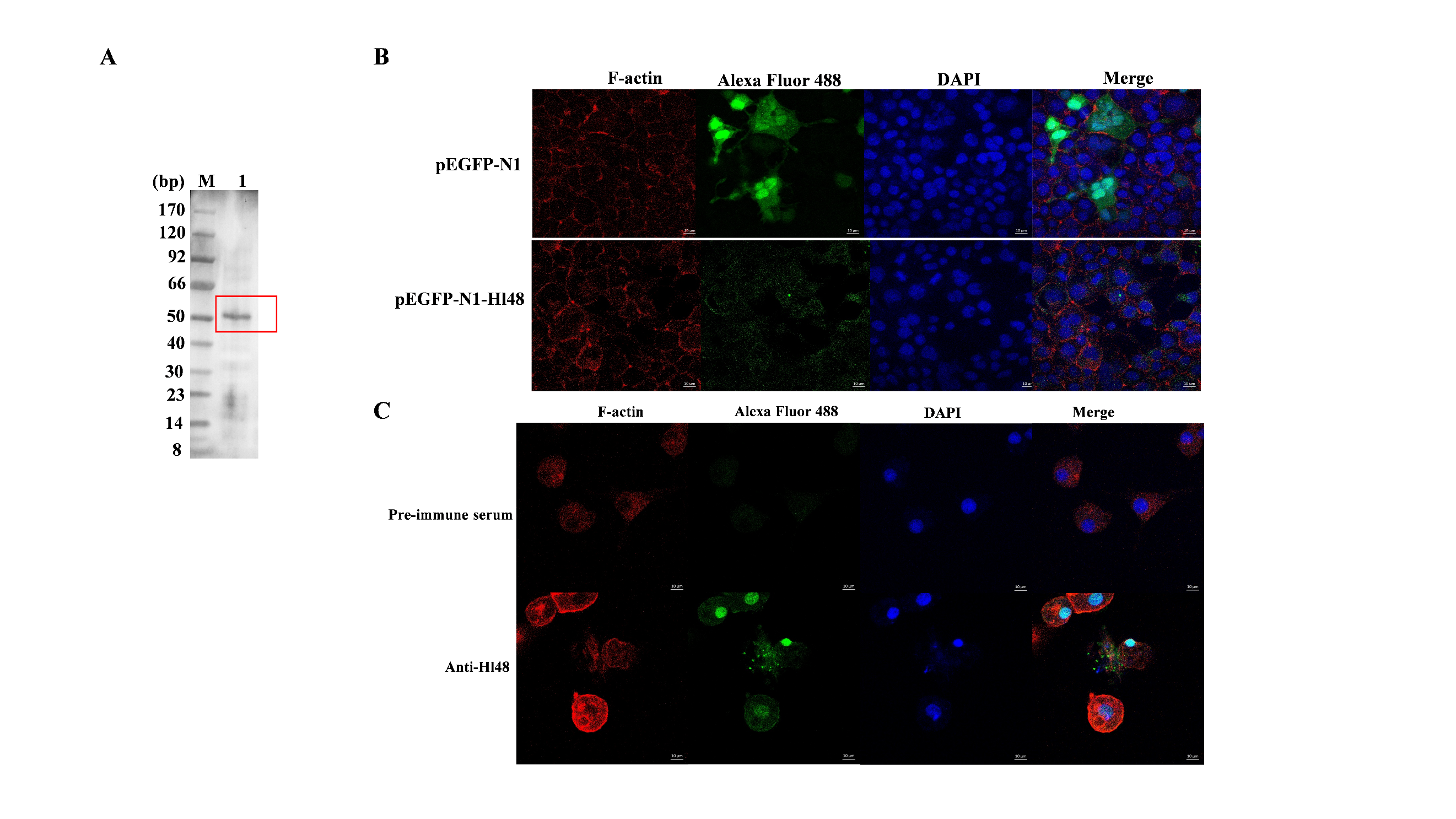


**Figure S2. Validation of Custom Hl48 Antibody and Subcellular localization of Hl48 in HEK293T cells.** (A)Verification of the anti-Hl48 antibody specificity using Western blot. (B-C) Subcellular localization of Hl48 in HEK293T cells (B) and IRE/CTVM19 cells (C). Cells were transfected with a Hl48-expressing plasmid for 48 hours and subjected to immunofluorescence staining. Hl48 (green) shows a predominantly localized in the cytoplasm and nucleus. F-actin (red) and DAPI (blue) mark the cytoskeleton and nuclei, respectively. Scale bar, 10 μm.


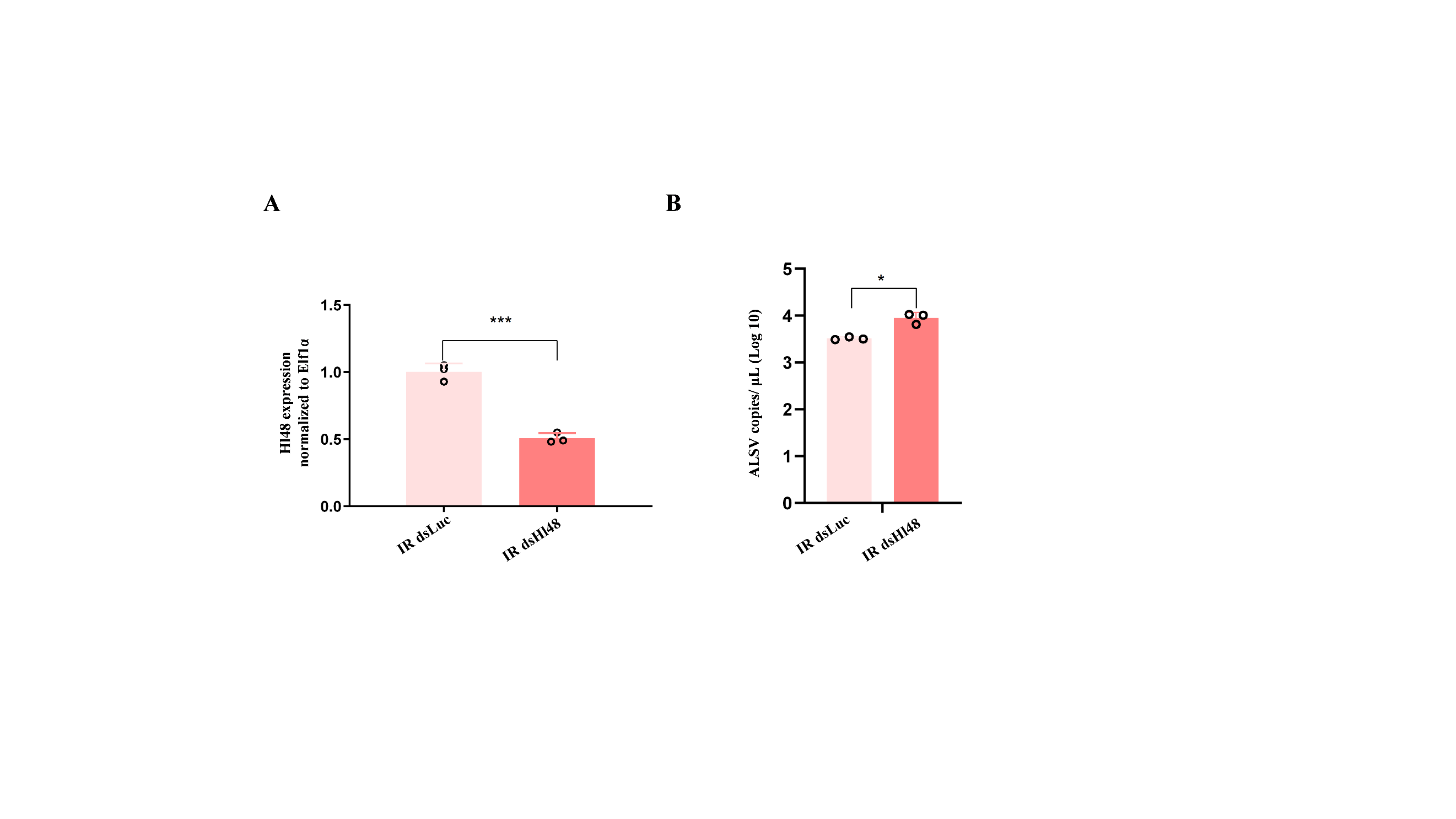


**Figure S3.** **Interference of Hl48 in tick cells impairs antiviral RNAi and promotes ALSV replication.** (A) RNAi efficiency of dsHl48 in cell line IRE/CTVM19. Cells were transfected with dsHl48 or dsLuc using RNAiMAX, and Hl48 mRNA levels were quantified by qRT-PCR at 48 h post-transfection. Data are presented as mean ± SD (n = 3). ***p < 0.001. (B) ALSV genomic copy numbers in IRE/CTVM19 cells following Hl48 knockdown. Cells transfected with dsHl48 or dsLuc were infected with ALSV at 48 h post-transfection. Viral RNA was extracted and copy numbers were determined by absolute quantification qRT-PCR using a standard curve. Results are expressed as ALSV copies per μL and presented on a log₁₀ scale. Data are presented as mean ± SD (n = 3). *p < 0.05.


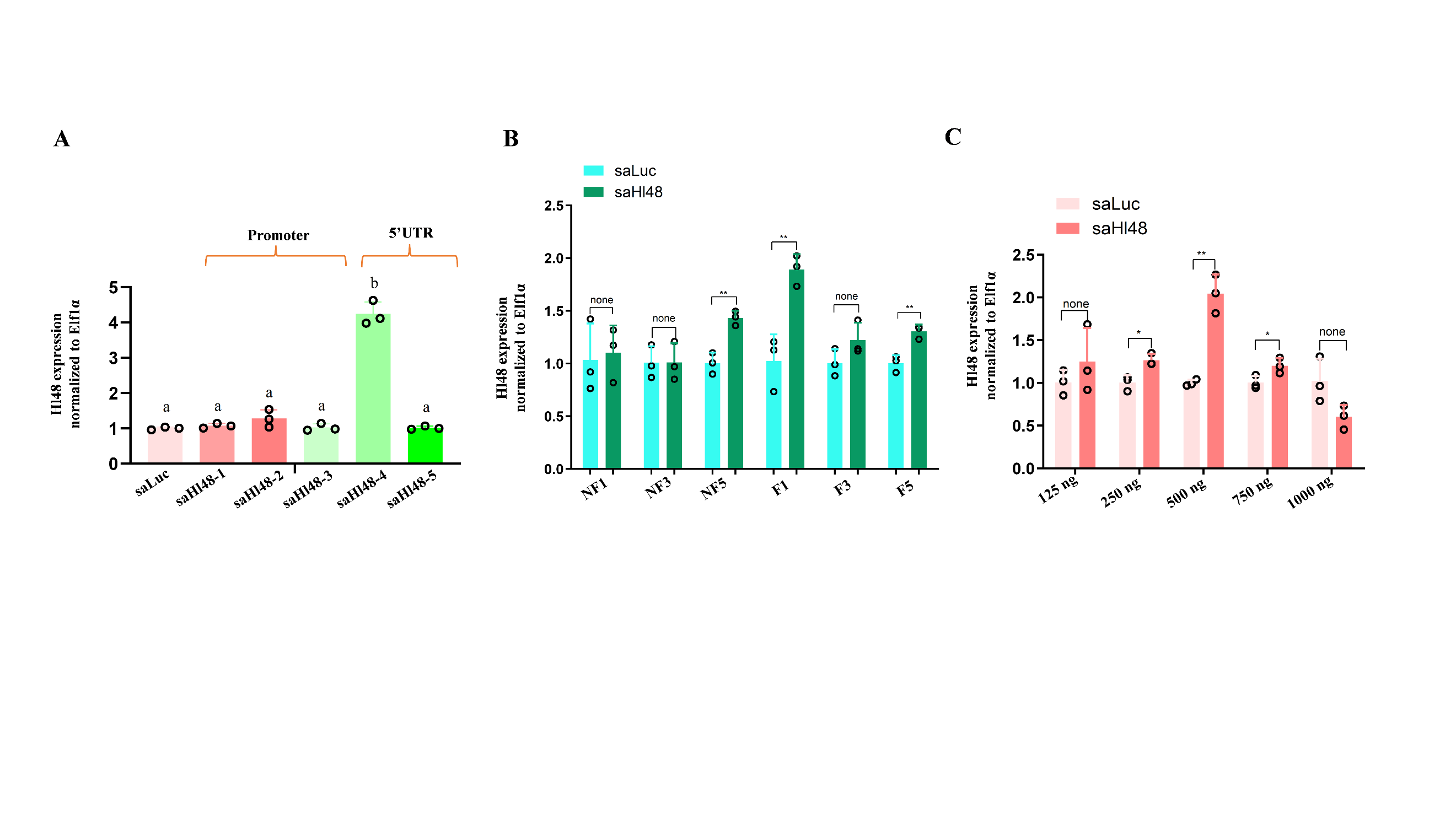


**Figure S4. validation and optimization of saHl48.** (A) Screening of candidate saRNA sequences targeting Hl48. Four candidate saRNAs were designed using two strategies: two based on predicted promoter regions upstream of the transcription start site (saRNA-1 and saRNA-2) and three based on the experimentally validated 5′ UTR sequence obtained by RACE (saRNA-3, saRNA-4 and saRNA-5). Hl48 mRNA levels were quantified by qRT-PCR in engorged adult whole ticks after saHl48 injection. (B) Time-course analysis of Hl48 mRNA levels in unfed and fed adult ticks after injection with saHl48. NF, unattached; F, attached. (C) Determination of the optimal injection dose for saHl48 by evaluating Hl48 expression in whole tick after administration of increasing amounts of saRNA. Data are presented as mean ± SD (n = 3). one-way ANOVA followed by Tukey's HSD post hoc test (A); Student's t-test (B, C). *p < 0.05, **p < 0.01, NONE, not significant.


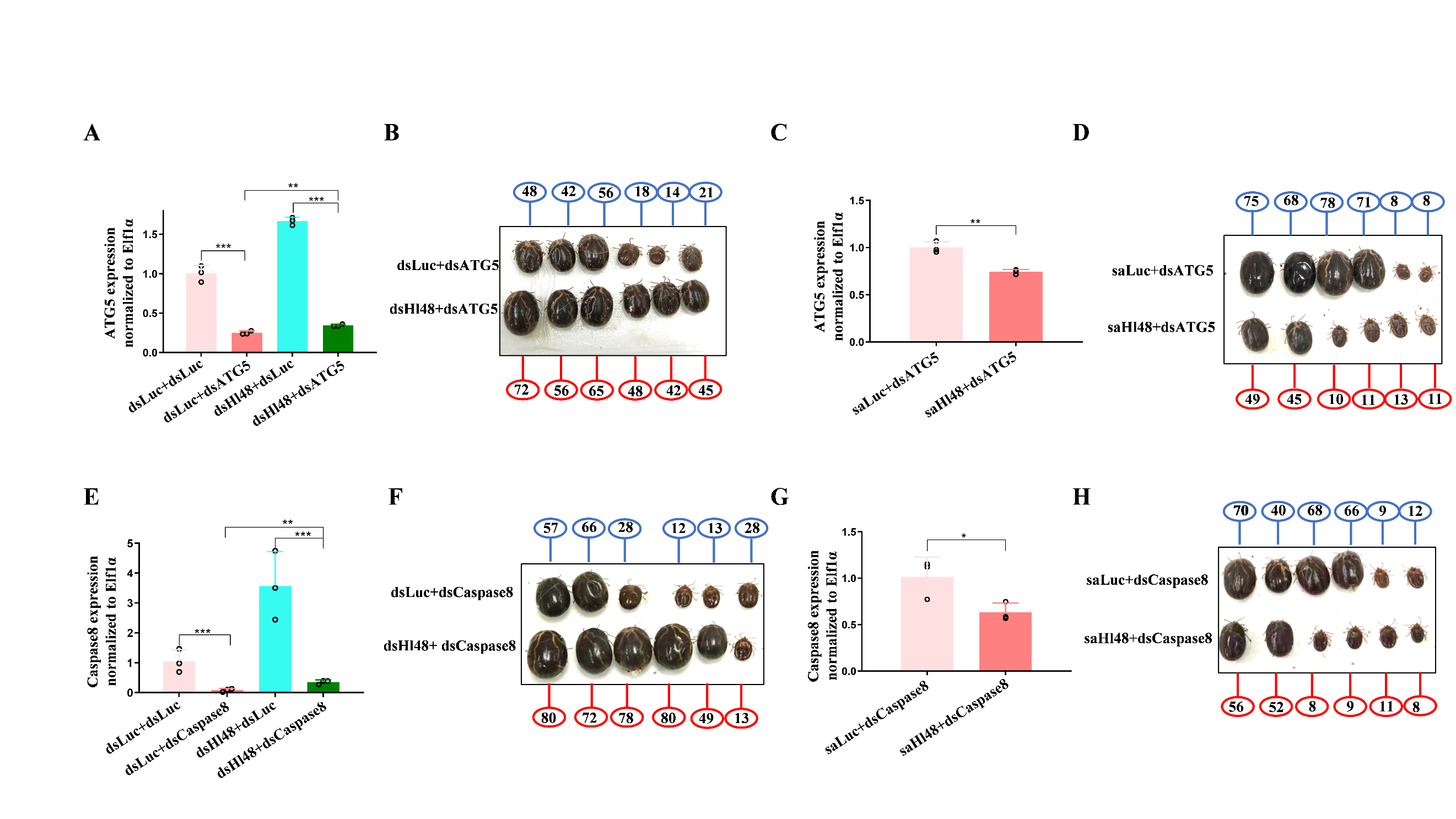


**Figure S5. Hl48 modulates RNAi efficiency of dsATG5 and dsCaspase8 in *H. longicornis*.** (A) qRT-PCR analysis of ATG5 mRNA levels in whole ticks pre-treated with dsLuc or dsHl48 followed by dsATG5 injection. (B) Representative images of engorged ticks from dsATG5 RNAi-of-RNAi experiments; numbers indicate individual body weight (mg). (C) qRT-PCR analysis of ATG5 mRNA levels in whole ticks following Hl48 activation and subsequent dsATG5 treatment. (D) Representative images of engorged ticks from dsATG5 RNAi-of-RNAa experiments. (E) qRT-PCR analysis of Caspase8 mRNA levels in whole ticks pre-treated with dsLuc or dsHl48 followed by dsCaspase8 injection. (F) Representative images of engorged ticks from dsCaspase8 RNAi-of-RNAi experiments; numbers indicate individual body weight (mg). (G) qRT-PCR analysis of Caspase8 mRNA levels in whole ticks following Hl48 activation and subsequent dsCaspase8 treatment. (H) Representative images of engorged ticks from dsCaspase8 RNAi-of-RNAa experiments. Data are presented as mean ± SD (n = 3). *p < 0.05, **p < 0.01, ***p < 0.001.


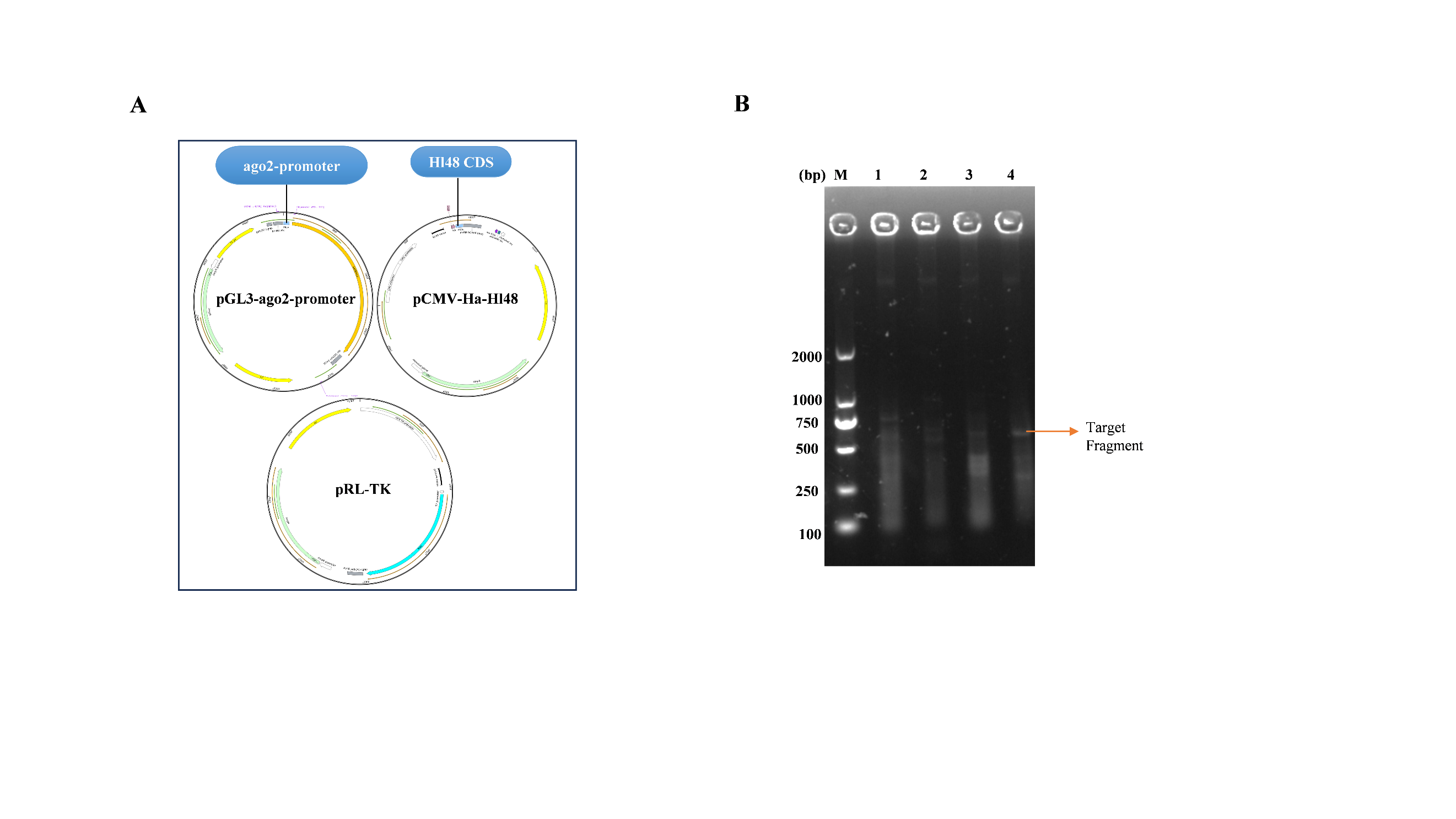


**Figure S6. Molecular Identification and Transcriptional Regulation Analysis of ago2.** (A) Schematic representation of dual-luciferase reporter constructs for assessing ago2 promoter activity. (B) Agarose gel electrophoresis of ago2 promoter using gene-specific primers. M. DNA marker; 1. 5’GSP-1; 2. 5’GSP-2; 3. 5’GSP-3; 4. 5’GSP-4.
