# Supplementary material for: Hl48 modulates argonaute 2 to enhance RNA interference in ticks
Source: Front Cell Infect Microbiol. 2026 Jun 5;16:1849245. doi: 10.3389/fcimb.2026.1849245 (PMC13280550; doi:10.3389/fcimb.2026.1849245)
Supplement: Supplementary file 1 [file DataSheet1.zip › supplementary table.docx]

**SUPPLEMENTARY TABLE**

**Table S1 PCR primers related to experimental procedures.**

| Name | Sequence (5’ to 3’) |
| --- | --- |
| Forward primer for full-length Hl48 | ATGTTCCAGTCTAAGTTTCTGCG |
| Reverse primer for full-length Hl48 | TCAGAGCTCGTCCCTCTCG |
| Forward primer for full-length *ago2* | ATGACCCAGCTGAGGGCAGCCAGGAG |
| Reverse primer for full-length *ago2* | TCAGACAAAGTACATGATCTTCTGCATC |
| Forward primer for *ago2*-piwi | ATGGGCGCACAATGCCTG |
| Reverse primer for *ago2*-piwi | GGTGATGTGCTCTTTGGCCC |
| 5’GSP for Hl48 5’UTR | CAATGGCCAGCAGGCCGCCCATG |
| 3’GSP for Hl48 3’UTR | AGACGTCCAGGAGAAGGTGGTGCAAGC |
| Forward primer 1 for Hl48 dsRNA | GGATCCTAATACGACTCACTATAGGCCAAGAAAGAGTTTGAAGC |
| Reverse primer 1 for Hl48 dsRNA | TGATGCCGAACCAAGGGTGAA |
| Forward primer 2 for Hl48 dsRNA | CCAAGAAAGAGTTTGAAGC |
| Reverse primer 2 for Hl48 dsRNA | GGATCCTAATACGACTCACTATAGGTGATGCCGAACCAAGGGTGAA |
| Forward primer 1 for Luc dsRNA | GGATCCTAATACGACTCACTATAGGGCTTCCATCTTCCAGGGATACG |
| Reverse primer 1 for Luc dsRNA | CGTCCACAAACACAACTCCTCC |
| Forward primer 2 for Luc dsRNA | GCTTCCATCTTCCAGGGATACG |
| Reverse primer 2 for Luc dsRNA | GGATCCTAATACGACTCACTATAGGCGTCCACAAACACAACTCCTCC |
| Forward primer 1 for ECR dsRNA | GGATCCTAATACGACTCACTATAGGGACAGCGAGGAAGACAACCA |
| Reverse primer 1 for ECR dsRNA | AAGCACATCTCGGCGTTCAT |
| Forward primer 2 for ECR dsRNA | GACAGCGAGGAAGACAACCA |
| Reverse primer 2 for ECR dsRNA | GGATCCTAATACGACTCACTATAGGAAGCACATCTCGGCGTTCAT |
| Forward primer 1 for ATG5 dsRNA | GGATCCTAATACGACTCACTATAGGAGGAATTTCCCGAGAAAC |
| Reverse primer 1 for ATG5 dsRNA | GGTCCATCACCAGACAGGAC |
| Forward primer 2 for ATG5 dsRNA | AGGAATTTCCCGAGAAAC |
| Reverse primer 2 for ATG5 dsRNA | GGATCCTAATACGACTCACTATAGGGGTCCATCACCAGACAGGAC |
| Forward primer 1 for Caspase8 dsRNA | GGATCCTAATACGACTCACTATAGGAGCGAGACGGTACCCG |
| Reverse primer 1 for Caspase8 dsRNA | GGTCCCGGTACGACAC |
| Forward primer 2 for Caspase8 dsRNA | AGCGAGACGGTACCCG |
| Reverse primer 2 for Caspase8 dsRNA | GGATCCTAATACGACTCACTATAGGGGTCCCGGTACGACAC |
| Forward primer 1 for IR Hl48 dsRNA | GGATCCTAATACGACTCACTATAGGAGGTCCTTCCACGTGGTGT |
| Reverse primer 1 for IR Hl48 dsRNA | TGCATGAGAGCCTCTTGAG |
| Forward primer 2 for IR Hl48 dsRNA | AGGTCCTTCCACGTGGTGT |
| Reverse primer 2 for IR Hl48 dsRNA | GGATCCTAATACGACTCACTATAGGTGCATGAGAGCCTCTTGAG |

**Table S2 qRT-PCR primers related to experimental procedures.**

| Name | Sequence (5’ to 3’) |
| --- | --- |
| Forward for Hl48 | TTCTGCGTCTTGTTGTGG |
| Reverse for Hl48 | CCTCCTCTTCCTTCTTGCT |
| Forward for ELF1α | CGTCTACAAGATTGGTGGCATT |
| Reverse for ELF1α | CTCAGTGGTCAGGTTGGCAG |
| Forward for ECR | CAGTGCAAGTACGGCAATAAC |
| Reverse for ECR | GACGCTGAGGCACTTCTT |
| Forward for ATG5 | GACCCCAGCACATGGTCA |
| Reverse for ATG5 | CTAAGAGCACGGCAGGAT |
| Forward for Caspase8 | CCCAAACGCATCAGCAAA |
| Reverse for Caspase8 | GCCACCTTCATAGAGCAACAC |
| Forward for *ago2* | CAAGAGAAGCCAGCCATCT |
| Reverse for *ago2* | CGAACTGTCCCTCGCTAACT |
| Forward for IR Hl48 | GCATTGCTGGTGCTCTTAG |
| Reverse for IR Hl48 | GTTCGTCCTCTTCATCTTCAG |
| *B. microti* -qPCR-F: | AACAGGCATTCGCCTTGAAT |
| *B. microt*i -qPCR-R: | CCAACTGCTCCTATTAACCATTACTCT |
| *B. microt*i -Probe | FAM-CTACAGCATGGAATAATGA-MGB |
| ALSV-S2-qPCR-F | GCTTGTGGTCATCATTATG |
| ALSV-S2-qPCR-R | CTCTGCCACATACTGATG |
| ALSV-S2 probe | FAM-CTCTCGTCAGCCATACCACCA-BHQ-1 |

**Table S3. Sequences of candidate saRNAs designed to target Hl48.**

| Name | Sequence (5’ to 3’) |
| --- | --- |
| saLuc | CGTACGCGGAATACTTCGA |
| saHl48-1 | GCAGAGCACAGGCATCTAA |
| saHl48-2 | GGACAGTACCATTAGTCAA |
| saHl48-3 | CATGTCAGGAGAAACACAA |
| saHl48-4 | GTGAGTCATTTGTGGTCTA |
| saHl48-5 | CCCGACAGTCTTGCAGCAA |

**Table S4 Confirmation of pathogen infection in ticks by quantitative PCR. CT values of ALSV and *B. microti* detected in tick samples. "/" indicates uninfected control groups.**

| groups | CT value | | |
| --- | --- | --- | --- |
|  | 1 | 2 | 3 |
| CTVM19 | / | / | / |
| ALSV | 29.829 | 29.298 | 28.800 |
| Uninfected *B. microti* | / | / | / |
| *B. microti* | 21.266 | 22.182 | 22.873 |

**Table S5 Hl48 interference or a****ctivation affects the biological characteristics of *H. longicornis* adult.**

| groups | Engorgement rate (%) | | | Body weight (mg) | |
| --- | --- | --- | --- | --- | --- |
|  | Engorged ticks/Total ticks | Mean ± SD | P value | Median (Q1–Q3) | P value |
| dsLuc | 76/90 | 86.66 ± 3.34% | ns | 74 (66–78) | ns |
| dsHl48 | 77/90 | 85.55 ± 5.03% |  | 75.5 (64.25–79) |  |
| saLuc | 81/90 | 89.99 ± 8.74% | ns | 92 (79–96) | ns |
| saHl48 | 80/90 | 88.88 ± 5.09% |  | 86 (78–93) |  |

Data are presented as mean ± SD (n = 3, 30 ticks per replicate) for engorgement rate, and median (Q1–Q3) for body weight. Engorgement rates were compared using Fisher's exact test. Body weight was compared using Mann-Whitney U test. ns, not significant.

**Table S6 Hl48** **interference affects the role of other dsRNAs in the biological characteristics of *H. longicornis* adult.**

| groups | Engorgement rate (%) | | | Body weight (mg) | |
| --- | --- | --- | --- | --- | --- |
|  | Engorged ticks/Total ticks | Mean ± SD | P value | Median (Q1–Q3) | P value |
| dsLuc+dsECR | 0/90 | 0 ± 0% | ns | 12.5 (10–14.25) | **** |
| dsHl48+dsECR | 1/90 | 1.1 ± 1.9% |  | 17.0 (13–20.5) |  |
| dsLuc+dsATG5 | 34/90 | 37.78 ± 3.85% | ns | 65 (56–72) | * |
| dsHl48+dsATG5 | 44/90 | 49.0 ± 3.51% |  | 72 (65–79) |  |
| dsLuc+dsCaspase8 | 34/90 | 37.78 ± 5.09% | * | 66.5 (36.75–68.75) | **** |
| dsHl48+dsCaspase8 | 50/90 | 55.56 ± 3.85% |  | 89 (79–98) |  |

Same as Table S5

**Table S7 Hl48 activation affects the role of other dsRNAs in the biological characteristics of *H. longicornis* adult.**

| groups |  | Engorgement rate (%) | | Body weight (mg) | |
| --- | --- | --- | --- | --- | --- |
|  | Engorged ticks/Total ticks | Mean ± SD | P value | Median (Q1–Q3) | P value |
| saLuc+dsECR | 0/90 | 0 ± 0% | / | 13 (12–15) | **** |
| saHl48+dsECR | 0/90 | 0 ± 0% |  | 7 (6.5–7.5) |  |
| saLuc+dsATG5 | 39/90 | 43.89 ± 3.51% | * | 68 (56–75) | * |
| saHl48+dsATG5 | 23/90 | 25.00 ± 9.18% |  | 55 (43.5–65) |  |
| saLuc+dsCaspase8 | 37/90 | 41.67 ± 7.09% | * | 75.5 (63.25–80.25) | ** |
| saHl48+dsCaspase8 | 22/90 | 23.89 ± 3.51% |  | 60 (21.5–68.5) |  |

Same as Table S5

**Table S8 Analysis of the specific interaction sites between Hl48 and the AGO2-PIWI domain.** **The bold marked position is the mutation site.**

| Hl48 protein amino acid residue | HL-AGO2-PIWI protein amino acid residue | Distance (Å) |
| --- | --- | --- |
| VAL-10 | GLU-103 | 2.9, 3.6 |
| ALA-12 | ARG-104, ILE-106 | 3.1, 3.2 |
| VAL-14 | ILE-106 | 3.3 |
| **THR-28** | **ALA-29** | **2.2** |
| LYS-135 | ASP-180, ASP-220 | 3.2, 3.0 |
| **GLU-93** | **ARG-169** | **2.0, 2.4** |
| ASP-173 | ARG-157, HIS-155 | 3.2, 3.2, 2.7. 3.1 |
| GLU-148 | HIS-155 | 3.2 |
| GLN-180 | ARG-153 | 3.4 |
| ASP-182 | GLY-115 | 2.6 |
| ARG-183 | GLU-114 | 3.5 |
| LYS-350 | HIS-259 | 2.9 |

**Table S9 Kinetic parameters of the interaction between Hl48 protein and the AGO2-PIWI peptide determined by Biacore SPR.**

| **Ligand** | **Analyte** | **K_D_（M）** | **K_a_（1/Ms）** | **K_d_(1/s)** | **Rmax** |
| --- | --- | --- | --- | --- | --- |
| Hl48 protein | AGO2-PIWI peptide | 2.64E-06 | 647.1 | 0.001707 | 207.2 |
