# Supplementary material for: Hl48 modulates argonaute 2 to enhance RNA interference in ticks
Source: Front Cell Infect Microbiol. 2026 Jun 5;16:1849245. doi: 10.3389/fcimb.2026.1849245 (PMC13280550; doi:10.3389/fcimb.2026.1849245)
Supplement: Supplementary file 1 [file DataSheet1.zip › supplementary table.pdf]

SUPPLEMENTARY TABLE

Table S1 PCR primers related to experimental procedures.

Name	Sequence (5’ to 3’)
Forward primer for full-length Hl48	ATGTTCCAGTCTAAGTTTCTGCG
Reverse primer for full-length Hl48	TCAGAGCTCGTCCCTCTCG
Forward primer for full-length *ago2*	ATGACCCAGCTGAGGGCAGCCAGGAG
Reverse primer for full-length *ago2*	TCAGACAAAGTACATGATCTTCTGCATC
Forward primer for *ago2*-piwi	ATGGGCGCACAATGCCTG
Reverse primer for *ago2*-piwi	GGTGATGTGCTCTTTGGCCC
5’GSP for Hl48 5’UTR	CAATGGCCAGCAGGCCGCCCATG
3’GSP for Hl48 3’UTR	AGACGTCCAGGAGAAGGTGGTGCAAGC
Forward primer 1 for Hl48 dsRNA	GGATCCTAATACGACTCACTATAGGCCAAGAAAGAGTTTGAAGC
Reverse primer 1 for Hl48 dsRNA	TGATGCCGAACCAAGGGTGAA
Forward primer 2 for Hl48 dsRNA	CCAAGAAAGAGTTTGAAGC
Reverse primer 2 for Hl48 dsRNA	GGATCCTAATACGACTCACTATAGGTGATGCCGAACCAAGGGTGAA
Forward primer 1 for Luc dsRNA	GGATCCTAATACGACTCACTATAGGGCTTCCATCTTCCAGGGATACG
Reverse primer 1 for Luc dsRNA	CGTCCACAAACACAACTCCTCC
Forward primer 2 for Luc dsRNA	GCTTCCATCTTCCAGGGATACG
Reverse primer 2 for Luc dsRNA	GGATCCTAATACGACTCACTATAGGCGTCCACAAACACAACTCCTCC
Forward primer 1 for ECR dsRNA	GGATCCTAATACGACTCACTATAGGGACAGCGAGGAAGACAACCA
Reverse primer 1 for ECR dsRNA	AAGCACATCTCGGCGTTCAT
Forward primer 2 for ECR dsRNA	GACAGCGAGGAAGACAACCA
Reverse primer 2 for ECR dsRNA	GGATCCTAATACGACTCACTATAGGAAGCACATCTCGGCGTTCAT
Forward primer 1 for ATG5 dsRNA	GGATCCTAATACGACTCACTATAGGAGGAATTTCCCGAGAAAC
Reverse primer 1 for ATG5 dsRNA	GGTCCATCACCAGACAGGAC
Forward primer 2 for ATG5 dsRNA	AGGAATTTCCCGAGAAAC
Reverse primer 2 for ATG5 dsRNA	GGATCCTAATACGACTCACTATAGGGGTCCATCACCAGACAGGAC
Forward primer 1 for Caspase8 dsRNA	GGATCCTAATACGACTCACTATAGGAGCGAGACGGTACCCG
Reverse primer 1 for Caspase8 dsRNA	GGTCCCGGTACGACAC
Forward primer 2 for Caspase8 dsRNA	AGCGAGACGGTACCCG
Reverse primer 2 for Caspase8 dsRNA	GGATCCTAATACGACTCACTATAGGGGTCCCGGTACGACAC
Forward primer 1 for IR Hl48 dsRNA	GGATCCTAATACGACTCACTATAGGAGGTCCTTCCACGTGGTGT
Reverse primer 1 for IR Hl48 dsRNA	TGCATGAGAGCCTCTTGAG
Forward primer 2 for IR Hl48 dsRNA	AGGTCCTTCCACGTGGTGT
Reverse primer 2 for IR Hl48 dsRNA	GGATCCTAATACGACTCACTATAGGTGCATGAGAGCCTCTTGAG

Table S2 qRT-PCR primers related to experimental procedures.

Name	Sequence (5’ to 3’)
Forward for Hl48	TTCTGCGTCTTGTTGTGG
Reverse for Hl48	CCTCCTCTTCCTTCTTGCT
Forward for ELF1α	CGTCTACAAGATTGGTGGCATT
Reverse for ELF1α	CTCAGTGGTCAGGTTGGCAG

Forward for ECR	CAGTGCAAGTACGGCAATAAC
Reverse for ECR	GACGCTGAGGCACTTCTT
Forward for ATG5	GACCCCAGCACATGGTCA
Reverse for ATG5	CTAAGAGCACGGCAGGAT
Forward for Caspase8	CCCAAACGCATCAGCAAA
Reverse for Caspase8	GCCACCTTCATAGAGCAACAC
Forward for *ago2*	CAAGAGAAGCCAGCCATCT
Reverse for *ago2*	CGAACTGTCCCTCGCTAACT
Forward for IR Hl48	GCATTGCTGGTGCTCTTAG
Reverse for IR Hl48	GTTCGTCCTCTTCATCTTCAG
B. microti -qPCR-F:	AACAGGCATTCGCCTTGAAT
*B. microt*i -qPCR-R:	CCAACTGCTCCTATTAACCATTACTCT
*B. microt*i -Probe	FAM-CTACAGCATGGAATAATGA-MGB
ALSV-S2-qPCR-F	GCTTGTGGTCATCATTATG
ALSV-S2-qPCR-R	CTCTGCCACATACTGATG
ALSV-S2 probe	FAM-CTCTCGTCAGCCATACCACCA-BHQ-1

Table S3. Sequences of candidate saRNAs designed to target Hl48.

Name	Sequence (5' to 3')
saLuc	CGTACGCGGAATACTTCGA
saHl48-1	GCAGAGCACAGGCATCTAA
saHl48-2	GGACAGTACCATTAGTCAA
saHl48-3	CATGTCAGGAGAAACACAA
saHl48-4	GTGAGTCATTTGTGGTCTA
saHl48-5	CCCGACAGTCTTGCAGCAA

Table S4 Confirmation of pathogen infection in ticks by quantitative PCR. CT values of ALSV and *B. microti* detected in tick samples. "/" indicates uninfected control groups.

groups	CT value		
	1	2	3
CTVM19	/	/	/
ALSV	29.829	29.298	28.800
Uninfected *B. microti*	/	/	/
B. microti	21.266	22.182	22.873

Table S5 Hl48 interference or activation affects the biological characteristics of *H. longicornis* adult.

groups	Engorgement rate (%)			Body weight (mg)	
	Engorged ticks/Total ticks	Mean ± SD	P value	Median (Q1–Q3)	P value
dsLuc	76/90	86.66 ± 3.34%	ns	74 (66–78)	ns
dsHl48	77/90	85.55 ± 5.03%		75.5 (64.25–79)	
saLuc	81/90	89.99 ± 8.74%	ns	92 (79–96)	ns
saHl48	80/90	88.88 ± 5.09%		86 (78–93)	

Data are presented as mean ± SD (n = 3, 30 ticks per replicate) for engorgement rate, and median (Q1–Q3) for body weight. Engorgement rates were compared using Fisher's exact test. Body weight was compared using Mann-Whitney U test. ns, not significant.

Table S6 Hl48 interference affects the role of other dsRNAs in the biological characteristics of *H. longicornis* adult.

groups	Engorgement rate (%)			Body weight (mg)	
	Engorged ticks/Total ticks	Mean ± SD	P value	Median (Q1–Q3)	P value
dsLuc+dsECR	0/90	0 ± 0%	ns	12.5 (10–14.25)	****
dsHl48+dsECR	1/90	1.1 ± 1.9%		17.0 (13–20.5)	
dsLuc+dsATG5	34/90	37.78 ± 3.85%	ns	65 (56–72)	*
dsHl48+dsATG5	44/90	49.0 ± 3.51%		72 (65–79)	
dsLuc+dsCaspase8	34/90	37.78 ± 5.09%	*	66.5 (36.75–68.75)	****
dsHl48+dsCaspase8	50/90	55.56 ± 3.85%		89 (79–98)	

Same as Table S5

Table S7 Hl48 activation affects the role of other dsRNAs in the biological characteristics of *H. longicornis* adult.

groups		Engorgement rate (%)		Body weight (mg)	
	Engorged ticks/Total ticks	Mean ± SD	P value	Median (Q1–Q3)	P value
saLuc+dsECR	0/90	0 ± 0%	/	13 (12–15)	****
saHl48+dsECR	0/90	0 ± 0%		7 (6.5–7.5)	
saLuc+dsATG5	39/90	43.89 ± 3.51%	*	68 (56–75)	*
saHl48+dsATG5	23/90	25.00 ± 9.18%		55 (43.5–65)	
saLuc+dsCaspase8	37/90	41.67 ± 7.09%	*	75.5 (63.25–80.25)	**
saHl48+dsCaspase8	22/90	23.89 ± 3.51%		60 (21.5–68.5)	

Same as Table S5

Table S8 Analysis of the specific interaction sites between Hl48 and the AGO2-PIWI domain. The bold marked position is the mutation site.

Hl48 protein amino acid residue	HL-AGO2-PIWI protein amino acid residue	Distance (Å)
VAL-10	GLU-103	2.9, 3.6
ALA-12	ARG-104, ILE-106	3.1, 3.2
VAL-14	ILE-106	3.3
**THR-28**	**ALA-29**	**2.2**
LYS-135	ASP-180, ASP-220	3.2, 3.0
**GLU-93**	**ARG-169**	**2.0, 2.4**

ASP-173	ARG-157, HIS-155	3.2, 3.2, 2.7. 3.1
GLU-148	HIS-155	3.2
GLN-180	ARG-153	3.4
ASP-182	GLY-115	2.6
ARG-183	GLU-114	3.5
LYS-350	HIS-259	2.9

Table S9 Kinetic parameters of the interaction between Hl48 protein and the AGO2-PIWI peptide determined by Biacore SPR.

**Ligand**	**Analyte**	**K_D（M）**	**K_a（1/Ms）**	**K_d(1/s)**	**Rmax**
Hl48 protein	AGO2-PIWI peptide	2.64E-06	647.1	0.001707	207.2
